# Circadian Clock Control of Translation Initiation Factor eIF2α Activity Requires eIF2γ-Dependent Recruitment of Rhythmic PPP-1 Phosphatase in *Neurospora crassa*

**DOI:** 10.1128/mBio.00871-21

**Published:** 2021-05-18

**Authors:** Zhaolan Ding, Teresa M. Lamb, Ahmad Boukhris, Rachel Porter, Deborah Bell-Pedersen

**Affiliations:** a Department of Biology and Center for Biological Clocks Research, Texas A&M University, College Station, Texas, USA; Karlsruhe Institute of Technology

**Keywords:** eIF2α, PPP-1, phosphatase, translation initiation, circadian clock, eIF2γ, *Neurospora crassa*

## Abstract

The circadian clock controls the phosphorylation and activity of eukaryotic translation initiation factor 2α (eIF2α). In Neurospora crassa, the clock drives a daytime peak in the activity of the eIF2α kinase CPC-3, the homolog of yeast and mammalian GCN2 kinase. This leads to increased levels of phosphorylated eIF2α (P-eIF2α) and reduced mRNA translation initiation during the day. We hypothesized that rhythmic eIF2α activity also requires dephosphorylation of P-eIF2α at night by phosphatases. In support of this hypothesis, we show that mutation of N. crassa PPP-1, a homolog of the yeast eIF2α phosphatase GLC7, leads to high and arrhythmic P-eIF2α levels, while maintaining core circadian oscillator function. PPP-1 levels are clock-controlled, peaking in the early evening, and rhythmic PPP-1 levels are necessary for rhythmic P-eIF2α accumulation. Deletion of the N terminus of N. crassa eIF2γ, the region necessary for eIF2γ interaction with GLC7 in yeast, led to high and arrhythmic P-eIF2α levels. These data supported that N. crassa eIF2γ functions to recruit PPP-1 to dephosphorylate eIF2α at night. Thus, in addition to the activity of CPC-3 kinase, circadian clock regulation of eIF2α activity requires dephosphorylation by PPP-1 phosphatase at night. These data show how the circadian clock controls the activity a central regulator of translation, critical for cellular metabolism and growth control, through the temporal coordination of phosphorylation and dephosphorylation events.

## INTRODUCTION

The endogenous circadian clock is a conserved mechanism that allows organisms to anticipate daily environmental changes to maximize fitness (1–4). As such, it is linked to environmental sensing pathways that monitor external light, temperature, and nutrient availability. These input pathways provide information to modulate the clock. In turn, the clock utilizes feedback loops to sustain endogenous molecular oscillations and to generate rhythms in downstream output pathways, even in the absence of external cues (5–10). One of the most studied output pathways is rhythmic transcription, with up to 50% of the eukaryotic genome regulated by clock at the transcriptional level (9, 11–17). Furthermore, several transcript-modifying processes (including mRNA capping, splicing, polyadenylation, and deadenylation) are under clock control (11, 18–21). While mRNA rhythms contribute to the generation of rhythmic protein abundance, approximately 40 to 50% of rhythmic proteins in both mouse liver and in the fungus N. crassa derive from mRNAs that are not rhythmic (22–25), suggesting circadian regulation of protein stability and/or mRNA translation. In support of clock control of mRNA translation, the expression and/or phosphorylation of several translation factors are rhythmic in eukaryotic cells (22, 26–28), including rhythms in the phosphorylation and activity of the highly conserved translation initiation factor eIF2α (29–31). Interestingly, many of the proteins in this class (rhythmic protein, arrhythmic mRNA) revealed a metabolic time of day partitioning, with daytime peaks in proteins involved in catabolism or energy utilization and nighttime peaks in anabolism or energy storage (25). These findings support that clock control of translation impacts the metabolic state of the cell. Thus, understanding the connection between the clock and control of the energetically expensive process of translation is crucial for a complete understanding of cellular growth control.

How the clock controls translation is just beginning to be unraveled, with recent studies revealing a conserved role for the clock in control of translation initiation (29–31). Translation initiation starts with the formation of the ternary complex, which contains initiation factor eIF2, composed of α, β and γ subunits, Met-tRNA_i_^Met^ and GTP (32, 33). There are multiple steps in the process, but initiation ends and elongation begins when eIF5 mediates the hydrolysis of GTP-eIF2α to GDP-eIF2α, which along with the other initiation factors, dissociates and allows 40S- and 60S-ribosomal subunit joining to create the translation-competent 80S ribosome (34, 35). To initiate another round of translation, the guanine nucleotide exchange factor (GEF) eIF2B must charge GDP-eIF2α with GTP in a recycling step that is critical for controlling overall translation rates (32, 33). Ser51 phosphorylated eIF2α (P-eIF2α) inhibits eIF2B GEF activity by competitively binding to the limiting eIF2B (33), thus leading to reduced translation initiation of many mRNAs (32, 33), while also promoting translation of mRNAs with special motifs, including upstream ORFs (uORFs) (36). The levels of P-eIF2α have been correlated with cell growth, cancer, memory and learning and are stimulated by the integrated stress response (ISR) and the mammalian target of rapamycin (mTOR) pathways (37–39). In mammals (29, 30) and N. crassa (31), the circadian clock controls rhythms in P-eIF2α abundance. Thus, studies examining the interplay between the clock and the known input pathways will help reveal the full range of translational regulation by eIF2α phosphorylation.

The mechanism of clock control of eIF2α phosphorylation is currently best understood in the fungus N. crassa where the activity of the ISR responsive kinase CPC-3 (the homolog of yeast/mammalian GCN2) is thought to be modulated at different times of day via GCN1-dependent delivery of rhythmic levels of uncharged tRNAs (31). CPC-3 is required for Ser51 phosphorylation of eIF2α (P-eIF2α), and hyperactivation of CPC-3 kinase activity, either by pharmacological induction (3-AT) or by a constitutively active mutation (*cpc-3^c^*), abolished P-eIF2α rhythms. However, it is not known whether CPC-3 is sufficient to drive rhythms in P-eIF2α accumulation (31). In particular, we were interested in learning how P-eIF2α is converted back to the initiation competent dephosphorylated eIF2α and whether a phosphatase might also contribute to the daily rhythms in eIF2α activity.

Protein phosphatase 1 (PP1) dephosphorylates eIF2α in yeast (40) and mammalian (41) cells. This activity requires the catalytic subunit GLC7 in yeast, as well as PP1α, PP1β, or PP1γ isoforms in mammalian cells, and also requires one or more noncatalytic regulatory subunits to target PP1 to P-eIF2α (42). In mammalian cells, the RVxF motif present on GADD34 (PPP1R15A) and CReP (PPP1R15B) recruits PP1 to dephosphorylate Ser51 on eIF2α (43, 44). GADD34 and/or CReP homologs are present in chickens, frogs, and zebrafish, and a degenerate ortholog was identified in *Drosophila* (45). In yeast cells, however, there is no GADD34 or CReP homolog, but instead, an N-terminal extension of eIF2γ contains an RVxF motif that recruits GLC7 to dephosphorylate Ser51 of eIF2α (45). N. crassa PPP-1 (NCU00043) is the homolog of yeast GLC7 and is essential for survival (46). PPP-1 was previously shown to dephosphorylate FRQ protein to regulate the pace of the circadian clock (47). However, it was not known whether PPP-1 also functions to dephosphorylate P-eIF2α and control rhythmic eIF2α activity in N. crassa. In this study, we show that dephosphorylation of eIF2α *in vitro* required PPP-1, that PPP-1 levels are clock-controlled with a peak during the subjective night, and that the rhythm in PPP-1 accumulation is necessary for cycling P-eIF2α levels. Our study further revealed that the N terminus of eIF2γ, which lacks a consensus RVxF motif, is required to recruit PPP-1 to dephosphorylate eIF2α and maintain robust P-eIF2α rhythmicity but is not required to maintain circadian clock function.

## RESULTS

### PPP-1 phosphatase reduces P-eIF2α levels.

**FIG 1:**
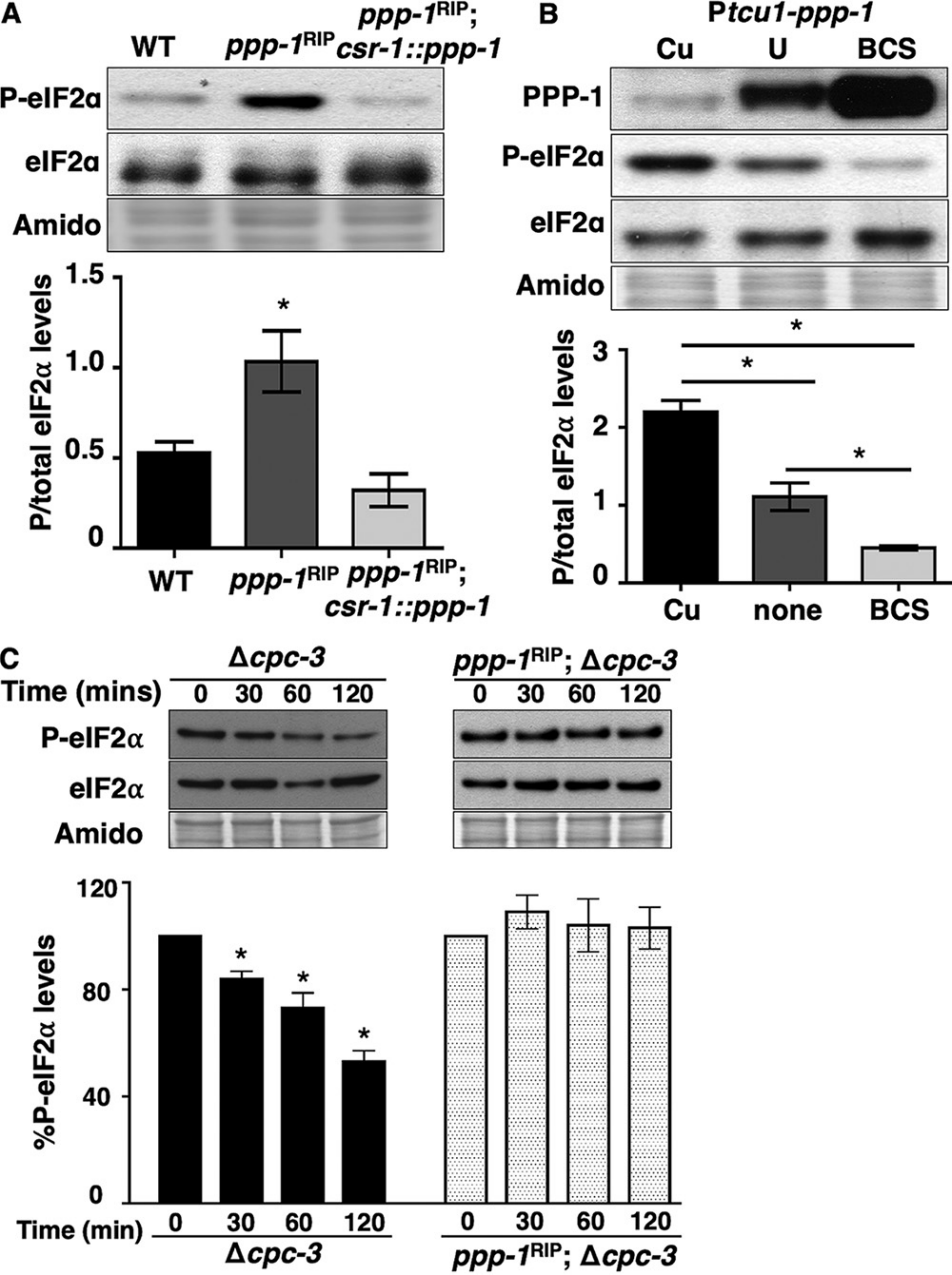
PPP-1 phosphatase reduces P-eIF2α levels and is critical for dephosphorylation of P-eIF2α *in vitro*. (A) Western blot of protein extracted from WT, *ppp-1^RIP^* mutant, and *ppp-1^RIP^*; *csr1*::*ppp-1* complemented strains during the subjective night (DD28) and probed with anti-P-eIF2α and total eIF2α antibodies. The P-eIF2α/total eIF2α signal is plotted below for each strain (mean ± the SEM, *n* = 3; *, *P* < 0.05 [Student's *t*-test]). (B) Western blot of protein from P*tcu1-ppp-1* cells grown in the presence of copper sulfate (Cu), BCS, or untreated (U); harvested at DD28; and probed with anti-PPP-1, anti-P-eIF2α, and anti-eIF2α antibodies. The graph below shows the average signal of P-eIF2α/total eIF2α (mean ± the SEM, *n* = 3; *, *P* < 0.05 [Student's *t*-test]). (C) *In vitro* dephosphorylation assay using cell extracts from Δ*cpc-3* and *ppp-1^RIP^*; Δ*cpc-3* cells incubated with P-eIF2α from *eIF2γ*::*v5* cells for 0, 30, 60, or 120 min. P-eIF2α and total eIF2α levels were examined by Western blotting. The graph below shows the average signal of P-eIF2α normalized to total protein for each time point and normalized to the value at time zero (mean ± the SEM, *n* = 4; *, *P* < 0.05 [Student's *t*-test compared to time zero]). In panels A to C, membranes were stained with amido black as a protein loading control.

To determine whether phosphatase PPP-1 regulates the levels of P-eIF2α in N. crassa, the levels and phosphorylation status of eIF2α were examined in *ppp-1^RIP^* mutant cells (47) from cultures grown in constant dark (DD) and harvested at 28 h (subjective night), which represents the low point of P-eIF2α abundance in wild-type (WT) cells (31) (Fig. 1A). P-eIF2α levels were significantly higher in *ppp-1^RIP^* mutant cells compared to WT cells harvested at 28 h, as well as in cells harvested in the subjective morning (DD40) (see Fig. S1A in the supplemental material) or grown in constant light (LL) (see Fig. S1B). The *ppp-1^RIP^* mutant was previously shown *in vitro* to reduce PPP-1 activity on P-phosphorylase by ∼70% (47). P-eIF2α abundance was ∼2-fold higher in *ppp-1^RIP^* cells compared to WT cells, suggesting that PPP-1 promotes the dephosphorylation of P-eIF2α. The abundance of total eIF2α was not altered in the *ppp-1^RIP^* cells (Fig. 1A). Complementation of *ppp-1^RIP^* cells with a WT copy of *ppp-1* inserted into the *csr-1* locus (*ppp-1^RIP^*; *csr-1*::*ppp-1*) reduced P-eIF2α levels to back to WT levels (Fig. 1A). These data support a role for PPP-1 in maintaining the low levels of P-eIF2α present at subjective night.

10.1128/mBio.00871-21.2 FIG S1 PPP-1 phosphatase reduces P-eIF2α levels. Western blots of protein extracted from WT and *ppp-1^RIP^* strains grown in DD and harvested during the subjective night (DD28), or subjective day (DD40) (A), or harvested after 24 h of growth in constant light (LL) (B) and probed with anti-P-eIF2α antibody. Membranes were stained with amido black as a protein loading control. The normalized P-eIF2α signal is plotted below (mean ± the SEM, *n* = 3; *, *P* < 0.05 [Student's *t*-test]). Download FIG S1, JPG file, 0.3 MB. Copyright © 2021 Ding et al. 2021 Ding et al. https://creativecommons.org/licenses/by/4.0/ This content is distributed under the terms of the Creative Commons Attribution 4.0 International license.

To determine whether changes in PPP-1 protein abundance can control P-eIF2α levels, *ppp-1* was put under the control of the copper regulatable P*tcu-1* promoter (48), and PPP-1 levels were detected using a PPP-1-specific antibody (see Fig. S2A). Consistent with the idea that PPP-1 controls P-eIF2α levels *in vivo*, copper sulfate (Cu) repression of P*tcu-1*::*ppp-1* led to low PPP-1 protein expression and high P-eIF2α levels. Conversely, addition of the copper chelator bathocuproinedisulfonic acid (BCS), led to high PPP-1 protein expression and low P-eIF2α levels compared to the control (U, untreated) (Fig. 1B). No significant changes were observed in eIF2α levels in any of the conditions used. Together, these data support the idea that PPP-1 either directly and/or indirectly reduces P-eIF2α levels in N. crassa.

10.1128/mBio.00871-21.3 FIG S2 Purification of PPP-1 and P-eIF2α, *in vitro* dephosphorylation of P-eIF2α, and interaction of PPP-1 with ribosomes. (A) Protein extracted from indicated *N. crassa* cells (*ppp-1^RIP^*, WT, and P*tcu1*::*ppp-1*) and *E.coli* cells expressing PPP-1::His6 were blotted with PPP-1 antibody. The expected molecular weight of PPP-1 protein is 35.8 kDa, and PPP-1::His6 is 36.8 kDa. (B) Coimmunoprecipitation (Co-IP) with anti-V5 in *eIF2γ*::*v5* cells pulls down P-eIF2α. V5 antibody was used to pull down protein from cell extracts of WT and *eIF2γ*::*V5* strains. Western blots were performed using the indicated antibodies. (C) *In vitro* dephosphorylation assay using only protein extraction buffer (Mock) and Δ*cpc-3* cell extracts (Δ*cpc-3*) incubated with P-eIF2α pulled down from *eIF2γ*::*v5* cells by anti-V5 IP for 0, 30, 60, or 120 mins. P-eIF2α and total eIF2α levels were examined by Western blotting. The graph below shows the P-eIF2α/total eIF2α signal relative to the value at time 0 (*n* = 1). (D) Cell extracts from WT cells harvested at DD24 were separated using sucrose density gradient (10 to 50%) centrifugation and fractionation. Western blots of the indicated fractions were probed with PPP-1 and control RPL3 antibodies. Membranes were stained with amido black as a protein loading control. Download FIG S2, JPG file, 1.2 MB. Copyright © 2021 Ding et al. 2021 Ding et al. https://creativecommons.org/licenses/by/4.0/ This content is distributed under the terms of the Creative Commons Attribution 4.0 International license.

To determine whether PPP-1 is responsible for dephosphorylating P-eIF2α, the eIF2 complex was purified from N. crassa cells containing a C-terminal V5-tagged eIF2γ (*eIF2γ*::*v5*) by coimmunoprecipitation with anti-V5 antibody (see Fig. S2B). To test whether there is a stable association of PPP-1 phosphatase with the eIF2 complex, we first examined P-eIF2α levels over time from the immunoprecipitated complex without addition of cell extract (Mock). We found that P-eIF2α levels in the mock treatments were unchanged over time, suggesting that PPP-1, or other phosphatases, were not copurified in the eIF2 complex (see Fig. S2C). Consistent with these data, PPP-1 was not detected in the eIF2 complex using anti-PPP-1 antibody in Western blots. To examine whether addition of PPP-1 can dephosphorylate P-eIF2α in the eIF2 complex, total protein extracts containing endogenous PPP-1 from subjective evening (DD28) cells were added. To avoid potential rephosphorylation by the CPC-3 kinase, we utilized *Δcpc-3* extracts (Fig. 1C). Cell extracts deficient in PPP-1 (*ppp-1^RIP^*; Δ*cpc-3*) were also examined (Fig. 1C). Extracts from Δ*cpc-3* cells led to an ∼50% reduction of P-eIF2α levels after 120 min, while no significant dephosphorylation of eIF2α was detected using extracts from *ppp-1^RIP^*; Δ*cpc-3* cells despite the *ppp-1^RIP^* mutant retaining some activity. These data suggested that the residual PPP-1 activity in the *ppp-1^RIP^* mutant is not sufficient to dephosphorylate P-eIF2α at a level that is detectable in *in vitro* assays. Taken together, these data support that *in vitro* dephosphorylation of P-eIF2α depends on the transient presence of PPP-1.

In S. cerevisiae, activation of the eIF2α kinase GCN2 *in vivo* requires its association with ribosomes (49). Uncharged tRNAs are transferred from the ribosome to GCN2 by GCN1 to activate GCN2 (50–53). We found that N. crassa PPP-1 associates with ribosomes (see Fig. S2D), suggesting the possibility that this interaction may facilitate direct access to its substrate P-eIF2α. Taken together, these results support the idea that PPP-1 promotes P-eIF2α dephosphorylation and are consistent with PPP-1 directly dephosphorylating eIF2α.

### PPP-1 phosphatase is required for clock control of P-eIF2α levels.

**FIG 2:**
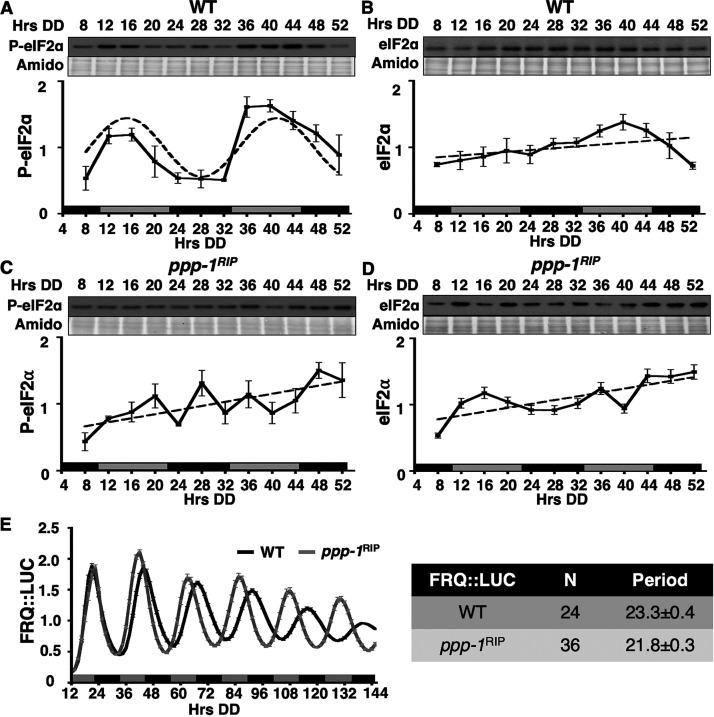
PPP-1 phosphatase is necessary for rhythmic P-eIF2α accumulation. Representative Western blots of protein isolated from WT (A and B) or *ppp-1^RIP^* (C and D) strains grown in a circadian time course, harvested at the indicated times in the dark (Hrs DD), and probed with anti-P-eIF2α antibody (A and C) or total eIF2α antibody (B and D). Membranes were stained with amido black as a protein loading control. Plots of the data (mean ± the SEM, *n* = 3) below show the average P-eIF2α (A and C) or eIF2α (B and D) signal normalized to total protein (solid line). Rhythmicity of P-eIF2α in WT cells (A) was determined by F-tests of fit to a sine wave (dotted line, *P* < 0.001), while P-eIF2α in *ppp-1^RIP^* cells (C) and eIF2α in WT (B) and *ppp-1^RIP^* cells (D) were arrhythmic, as shown by a better fit of the data to a line (dotted lines). The blots were probed separately and therefore cannot be used to compare protein levels between the strains. (E) Luciferase activity from a FRQ::LUC translational fusion in WT (black line) and *ppp-1^RIP^* (gray line) cells grown in DD and recorded every 90 min over 6 days (Hrs DD). The average normalized bioluminescence signal is plotted (mean ± the SEM, *n* = 24 for WT and 36 for *ppp-1^RIP^*). The period (h) (mean ± the SEM) of the FRQ::LUC rhythm is shown on the right and is significantly different between WT and *ppp-1^RIP^* (Student's *t*-test, *P* < 0.01).

To determine whether PPP-1 phosphatase controls rhythmic P-eIF2α levels in N. crassa, the phosphorylation status of eIF2α was examined in WT and *ppp-1^RIP^* cells grown in a circadian time course (Fig. 2). In WT cells, P-eIF2α, but not total eIF2α levels, were rhythmic, with a peak in the subjective late morning (Fig. 2A and B), consistent with our previous studies (31). While P-eIF2α and total eIF2α levels fluctuated in *ppp-1^RIP^* cells, P-eIF2α rhythms were abolished (Fig. 2C and D). Because the circadian clock was previously shown to be functional in *ppp-1^RIP^* cells (47), it seemed unlikely that P-eIF2α rhythms were abolished due to a clock defect in these cells. However, to confirm clock function in the mutant, FRQ::LUC protein rhythms were examined in WT and *ppp-1^RIP^* cells. Consistent with published data (47), FRQ levels oscillated robustly in *ppp-1^RIP^* cells, but with an ∼2 h shorter period compared to WT cells (Fig. 2E). Taken together, these data support the idea that the loss of P-eIF2α rhythms in *ppp-1^RIP^* cells is not due to loss of rhythmicity of the core oscillator, but instead results from disruption of downstream circadian regulation of P-eIF2α levels.

### Deletion of the N terminus of eIF2γ alters eIF2α phosphorylation levels and the dephosphorylation rate of eIF2α *in vitro*.

**FIG 3:**
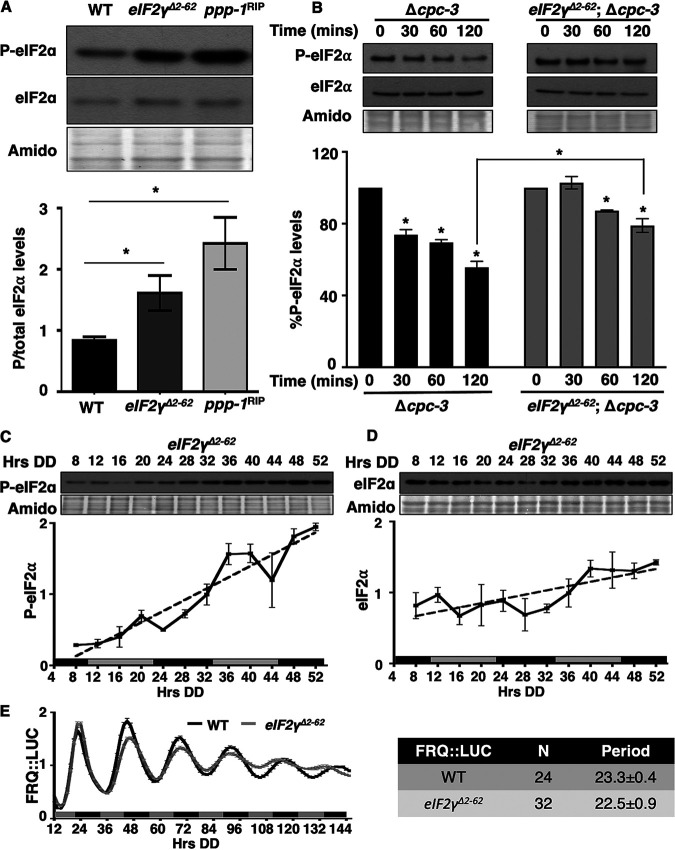
Deletion of the amino terminal 60 amino acids of N. crassa eIF2γ alters P-eIF2α levels and rhythmicity. (A) Western blot of protein extracted from the indicated strains harvested at DD28 were probed with anti-P-eIF2α or total eIF2α antibodies. P-eIF2α/total eIF2α signals are plotted below (mean ± the SEM, *n* = 3; *, *P* < 0.05 [Student's *t*-test]). (B) *In vitro* dephosphorylation assay using cell extracts from Δ*cpc-3* and *eIF2γ^Δ2-62^*; Δ*cpc-3* cells incubated with pulled down P-eIF2α from *eIF2γ*::*v5* and *eIF2γ^Δ2-62^*::*v5* cells, respectively, for 0, 30, 60, and 120 min. P-eIF2α and total eIF2α levels were examined by Western blotting. The graph below shows the average signal of P-eIF2α normalized to total protein for each time point and normalized to the value at time zero (mean ± the SEM, *n* = 5; *, *P* < 0.05 [Student's *t*-test compared with time zero]). (C and D) Western blots of protein from *eIF2γ^Δ2-62^* cells grown in a circadian time course, harvested at the indicated times in DD (Hrs DD), and probed with anti-P-eIF2α (C) or anti-total eIF2α (D) antibody. Plots of the data (mean ± the SEM, *n* = 5) below display the average P-eIF2α (C) or eIF2α (D) signal normalized to total protein (solid line). Both P-eIF2α and total eIF2α in *eIF2γ^Δ2-62^* cells were arrhythmic determined by F tests of the fit to a line (dotted lines). Membranes were stained with amido black as a protein loading control. (E) Luciferase activity from a FRQ::LUC translational fusion expressed in WT (black line) and *eIF2γ^Δ2-62^* (gray line) cells grown in DD and recorded every 90 min over 6 days (Hrs DD). The average normalized bioluminescence signal is plotted (mean ± the SEM, *n* = 24 for WT and *n* = 32 for *eIF2γ^Δ2-62^*). The period (h) (mean ± the SEM) is shown on the right.

The N terminus of N. crassa eIF2γ (NCU02810) resembles the N terminus of the S. cerevisiae eIF2γ in that it has an 80-amino-acid extension compared to eIF2γ homologs in higher eukaryotes. In S. cerevisiae this region is required to recruit PPP-1 to eIF2α (45) (see Fig. S3A). We predicted that if the N terminus of N. crassa eIF2γ functions analogously, the levels of P-eIF2α would be high in strains that have an N-terminal eIF2γ deletion. To test this prediction, residues 2 to 62 were deleted from the endogenous eIF2γ gene (here referred to as *eIF2γ^Δ2-62^*) (see Fig. S3A), and P-eIF2α levels were examined in a circadian time course (Fig. 3). As predicted, removal of this putative phosphatase-recruiting domain resulted in significantly higher P-eIF2α levels in *eIF2γ^Δ2-62^* compared to WT cells. Furthermore, P-eIF2α levels in *eIF2γ^Δ2-62^* cells were not significantly different than the high levels observed in *ppp-1*^RIP^ cells (Fig. 3A). These results support a role for the N-terminal region of N. crassa eIF2γ in recruiting PPP-1 phosphatase to P-eIF2α *in vivo*.

10.1128/mBio.00871-21.4 FIG S3 Comparison of protein domains of eIF2γ in N. crassa with S. cerevisiae and interaction of P-eIF2α with *eIF2γ*::*v5* (A) Schematic diagram shows the location of the GTP-binding (G) domain and domains II (DII) and III (DIII) of eIF2γ in N. crassa and S. cerevisiae based on domain information from InterPro (https://www.ebi.ac.uk/interpro/). The numbers mark the amino acids that make up the N-terminal extension. Below is alignment of the N-terminal amino acid sequence of eIF2γ in N. crassa with S. cerevisiae. The sequence marked with a star indicates the binding motif KKVAF of eIF2γ in S. cerevisiae. (B) Anti-V5 antibody was used in Co-IP reactions to pull down protein from cell extracts of WT, *eIF2γ*::*v5*, and *eIF2γ^Δ2-62^*::*v5* strains. Western blotting of input and IP samples was performed with the indicated antibodies. Download FIG S3, JPG file, 0.5 MB. Copyright © 2021 Ding et al. 2021 Ding et al. https://creativecommons.org/licenses/by/4.0/ This content is distributed under the terms of the Creative Commons Attribution 4.0 International license.

To determine whether the N terminus of eIF2γ impacts dephosphorylation of eIF2α by PPP-1 *in vitro*, the eIF2 complex was purified from N. crassa *eIF2γ*::*v5* and *eIF2γ^Δ2-62^*::*v5* cells by coimmunoprecipitation with anti-V5 antibody (see Fig. S3B). The eIF2 complex containing pulled down P-eIF2α with eIF2γ::V5 was incubated with total cell extracts (containing PPP-1) from Δ*cpc-3* cells, and the eIF2 complex pulled down with eIF2γ^Δ2-62^::V5 was incubated with total cell extracts from *eIF2γ^Δ2-62^*; Δ*cpc-3* cells (Fig. 3B). Extracts from Δ*cpc-3* cells led to an ∼50% reduction of P-eIF2α levels after 120 min, consistent with dephosphorylation of P-eIF2α by PPP-1 and the data shown in Fig. 1C. However, extracts from *eIF2γ^Δ2-62^*; Δ*cpc-3* cells that lack the N terminus of eIF2γ showed significantly reduced dephosphorylation of P-eIF2α levels compared to extracts from Δ*cpc-3* cells (Fig. 3B). In *eIF2γ^Δ2-62^*; Δ*cpc-3* cells, P-eIF2α levels were reduced up to 20% at 120 min compared to the 0-min time point, suggesting that additional regulatory subunits present in *eIF2γ^Δ2-62^*; Δ*cpc-3* extracts may recruit PPP-1 to dephosphorylate P-eIF2α, although less efficiently than eIF2γ. These results, together with the lack of dephosphorylation of P-eIF2α in mutant PPP-1 extracts (Fig. 1C), support the idea that the N terminus of eIF2γ recruits PPP-1 to dephosphorylate eIF2α in N. crassa.

### Deletion of the N terminus of eIF2γ disrupts P-eIF2α level rhythms.

To determine whether the N-terminal extension of eIF2γ is essential for circadian clock control of P-eIF2α, the levels of P-eIF2α were examined over a circadian time course in *eIF2γ^Δ2-62^* cells. The levels of P-eIF2α increased over time, and P-eIF2α rhythms were severely dampened in *eIF2γ^Δ2-62^* cells (Fig. 3C). When the data were detrended to account for the increasing levels of P-eIF2α over time, a rhythm with significantly reduced amplitude and period was detected (see Fig. S4). This dampened P-eIF2α rhythm observed in *eIF2γ^Δ2-62^* cells *in vivo* is consistent with residual PPP-1 phosphatase activity observed *in vitro* in *eIF2γ^Δ2-62^*; Δ*cpc-3* extracts (Fig. 3B). Total eIF2α levels in *eIF2γ^Δ2-62^* cells were arrhythmic (Fig. 3D; see also Fig. S4 in the supplemental material). Unlike the short period FRQ::LUC rhythm observed in *ppp-1^RIP^* cells (Fig. 2E), the period of FRQ::LUC reporter rhythms was not significantly altered in *eIF2γ^Δ2-62^* cells compared to WT cells (Fig. 3E). Therefore, it is likely that a different regulator is used to target PPP-1 to dephosphorylate FRQ. Also, PPP-1 protein levels were still rhythmic in *eIF2γ^Δ2-62^* cells, suggesting the mutation did not impact PPP-1 protein expression (see Fig. S4D). Taken together, these data support a role for the N terminus of eIF2γ in recruiting PPP-1 to P-eIF2α and promoting circadian clock control of P-eIF2α levels.

10.1128/mBio.00871-21.5 FIG S4 P-eIF2α rhythms are severely dampened in *eIF2γ^Δ2-62^* mutant cells. (A) P-eIF2α levels in WT (Fig. 2A) and *eIF2γ^Δ2-62^* (Fig. 3C) cells were plotted (WT solid black line; eIF*2γ^Δ2-62^* gray line) (mean ± the SEM, *n* = 5). The rhythmicity of P-eIF2α in WT cells was determined by F tests of fit to a sine wave (black dotted line, *P* < 0.001), while P-eIF2α in *eIF2γ^Δ2-62^* cells was arrhythmic as shown by a better fit of the data to a line (grey dotted lines). (B) Severely dampened rhythms were observed in P-eIF2α levels from *eIF2γ^Δ2-62^* cells when the data were detrended. A linear trendline was generated for P-eIF2α levels in WT and *eIF2γ^Δ2-62^* cells (Fig. 2A and 3C), and the differences between the raw data (mean) and the trendline are plotted. P-eIF2α levels in the detrended data were rhythmic in WT and *eIF2γ^Δ2-62^* cells as determined by F tests of the fit to sine waves (dotted lines), but with reduced significance in the mutant (*P* values provided in the table below); and with significantly reduced amplitude and period (*P* < 0.05 [Student's *t*-test]). The peak phase of the rhythm, measured in circadian time (CT) was not significantly different between WT and *eIF2γ^Δ2-62^* cells. (C) Total eIF2α levels in WT (Fig. 2B) and *eIF2γ^Δ2-62^* (Fig. 3D) cells were plotted (WT solid black line; eIF*2γ^Δ2-62^* gray line) (mean ± the SEM, *n* = 5). Dotted lines show the fit of the data to a line by F tests. (D) Deletion of the N-terminal extension does not impact PPP-1 protein rhythms. Luciferase activity from a PPP-1::LUC translational fusion expressed in WT (black line) and *eIF2γ^Δ2-62^* (grey line) cells grown in DD and recorded every 90 min over 6 days (Hrs DD). The average normalized bioluminescence signal is plotted (mean ± the SEM, *n* = 12). Download FIG S4, JPG file, 0.9 MB. Copyright © 2021 Ding et al. 2021 Ding et al. https://creativecommons.org/licenses/by/4.0/ This content is distributed under the terms of the Creative Commons Attribution 4.0 International license.

### Rhythmic phosphorylation of eIF2α requires rhythmic PPP-1 levels.

**FIG 4:**
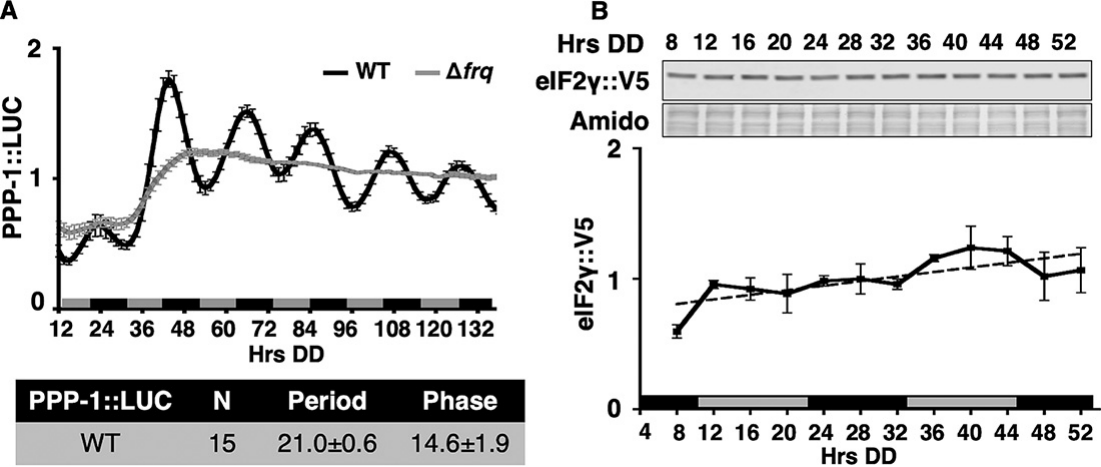
PPP-1 levels, but not *eIF2γ* levels, are controlled by the clock. (A) Luciferase activity from a PPP-1::LUC translational fusion in WT (black line) and Δ*frq* (gray line) cells grown in DD and recorded every 90 min over 6 days (Hrs DD). The average normalized bioluminescence signal is plotted (mean ± the SEM). Period and phase (h) (CT; mean ± the SEM) are shown below. (B) Western blot protein from *eIF2γ*::*v5* cells grown over a circadian time course, harvested at the indicated times (Hrs DD), and probed with anti-V5 antibody. Membranes were stained with amido black as a protein loading control. The average normalized signal is plotted below (mean ± the SEM) (solid black line). *eIF2γ*::*V5* levels were arrhythmic as indicated by the best fit of the data to a line (dotted line).

PPP-1 phosphatase and the N terminus of eIF2γ are necessary for circadian rhythms of P-eIF2α levels but not for core clock function. Thus, rhythmic control of eIF2α activity may be through clock control of the levels and/or activities of PPP-1 phosphatase and/or eIF2γ. Prior mass spectrometry proteomic studies suggested that PPP-1 protein, but not eIF2γ, could be rhythmic (25). To determine whether the circadian clock controls the levels of PPP-1 phosphatase and/or eIF2γ, PPP-1::luciferase (PPP-1::LUC) and eIF2γ::V5 C-terminal translational fusion constructs were generated and used to replace the corresponding endogenous loci. No change in P-eIF2α levels was observed in cells containing the V5-tagged version of eIF2γ::V5 compared to WT cells, indicating the tag does not alter the function of eIF2γ (see Fig. S5). PPP-1::LUC protein accumulated rhythmically in WT cells but not in control clock mutant Δ*frq* cells, (Fig. 4A), demonstrating that PPP-1 protein levels are clock-controlled. Consistent with PPP-1 functioning as an eIF2α phosphatase, the early evening peak (with phase CT [circadian time] 14, which corresponds to DD24) in PPP-1::LUC levels correlated with the trough of P-eIF2α levels (see Fig. 2A, DD24). Alternatively, eIF2γ::V5 levels did not cycle in WT cells (Fig. 4B). In addition, PPP-1::LUC rhythmicity was not altered in Δ*cpc-*3 cells that are unable to phosphorylate eIF2α (31) (see Fig. S6), indicating that PPP-1 protein level rhythms arise from mechanisms that are independent of rhythmic eIF2α activity. Together, these data suggested the possibility that the nighttime peak in PPP-1 levels may be critical for P-eIF2α rhythms.

10.1128/mBio.00871-21.6 FIG S5 Tagged eIF2γ::V5 is functional in N. crassa cells. Western blots of protein extracted from the indicated strains harvested at DD28 were probed with anti-P-eIF2α and total eIF2α antibodies. Membranes were stained with amido black as a protein loading control. The P-eIF2α/total eIF2α signal is plotted below (mean ± the SEM, *n* = 3). No significant differences in the levels of P-eIF2α were detected between WT and *eIF2γ*::*v5* strains (Student's *t*-test). Download FIG S5, JPG file, 0.1 MB. Copyright © 2021 Ding et al. 2021 Ding et al. https://creativecommons.org/licenses/by/4.0/ This content is distributed under the terms of the Creative Commons Attribution 4.0 International license.

10.1128/mBio.00871-21.7 FIG S6 Deletion of *cpc-3* does not impact PPP-1 protein rhythms. Luciferase activity from a PPP-1::LUC translational fusion expressed in WT (black line) and *Δcpc-3* (grey line) cells grown in DD and recorded every 90 min over 6 days (Hrs DD). The average normalized bioluminescence signal is plotted (mean ± the SEM). The period (h) (mean ± the SEM) is shown in the table. Download FIG S6, JPG file, 0.2 MB. Copyright © 2021 Ding et al. 2021 Ding et al. https://creativecommons.org/licenses/by/4.0/ This content is distributed under the terms of the Creative Commons Attribution 4.0 International license.

**FIG 5:**
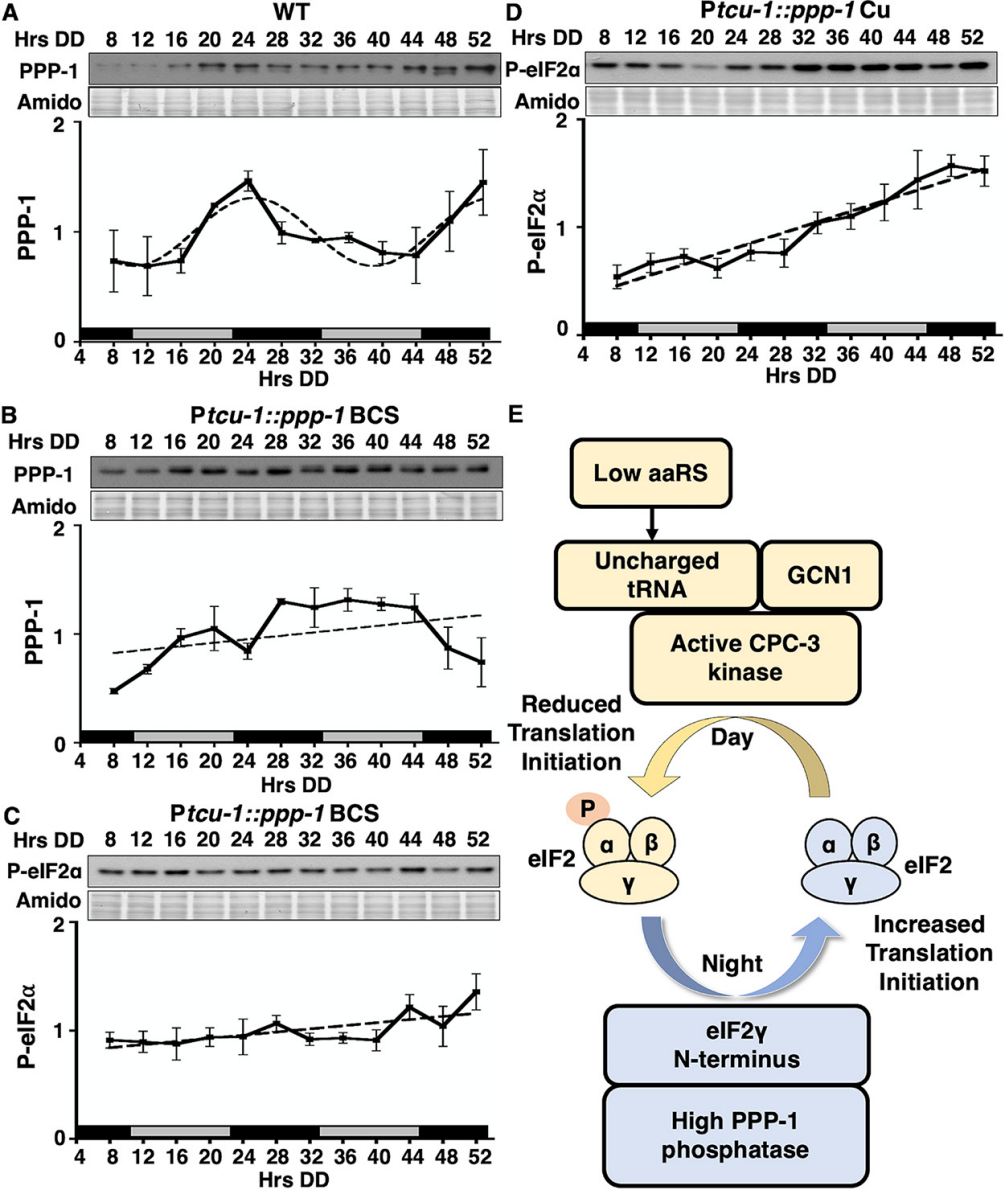
Clock control of PPP-1 is necessary for rhythmic P-eIF2α levels. (A to D) Western blots of protein extracted from WT (A) or P*tcu1*::*ppp-1* cells cultured with 50 μM BCS (B and C) or 250 μM copper sulfate (D) over a circadian time course, harvested at the indicated times in DD (Hrs DD), and probed with anti-PPP-1 (A and B) or anti-P-eIF2α (C and D) antibodies. Membranes were stained with amido black as a protein loading control. The normalized protein levels are plotted below the blots (mean ± the SEM, *n* = 3) (solid black line). PPP-1 levels in WT cells (A) were rhythmic based on best fit to a sine wave (dotted line, *P* < 0.001), whereas PPP-1 and P-eIF2α (B to D) were arrhythmic as indicated by best fit to a line (dotted line). (E) Model of the mechanisms of clock coordination of the day-active eIF2α kinase CPC-3 and the night-active phosphatase PPP-1 controlling rhythmic eIF2α activity and translation initiation.

To determine whether rhythmic accumulation of PPP-1 is necessary for rhythms in P-eIF2α levels, protein from strains containing *Ptcu-1*::*ppp-1* (Fig. 1B) grown in a circadian time course were isolated and examined by Western blotting with anti-PPP-1 antibody. In WT cells, PPP-1 protein levels were rhythmic, peaking in the subjective early evening (DD24), consistent with the PPP-1::LUC rhythms (Fig. 5A). In P*tcu1*::*ppp-1* cells grown in the presence of the activating chelator BCS, PPP-1 levels were high and noncycling (Fig. 1B and 5B), and P-eIF2α levels were low and arrhythmic (Fig. 5C). In P*tcu1*::*ppp-1* cells grown in the presence of the repressive copper ion (Cu), PPP-1 protein levels were low (Fig. 1B), and P-eIF2α levels were high and arrhythmic (Fig. 5D). Thus, nonrhythmic PPP-1 expression at either low or high levels abolished P-eIF2α rhythms. These data demonstrated that the rhythmic accumulation of PPP-1 protein is necessary for circadian rhythms in P-eIF2α levels.

## DISCUSSION

In N. crassa and mice, circadian clock regulation of eIF2α phosphorylation controls rhythmic mRNA translation and protein accumulation (30, 31). In N. crassa the eIF2α kinase, CPC-3, is necessary for the accumulation of P-eIF2α levels and a constitutively active allele causes arrhythmicity of P-eIF2α (31). Here, we show that protein phosphatase PPP-1, which peaks in levels during the subjective night, is also necessary for circadian rhythms in P-eIF2α levels. These data support a model whereby the circadian clock dynamically regulates both the phosphorylation, through the day-stimulated CPC-3 kinase, and dephosphorylation, by the night-peaking PPP-1 phosphatase, of eIF2α (Fig. 5E). The peak in activity of eIF2α at night, together with increased nighttime activity of translation elongation factor eEF-2 (28), provide a mechanism to explain increased rhythmic protein production at night in N. crassa (25).

While PPP-1 is necessary for rhythmic eIF2α activity, it is not sufficient to drive rhythms in P-eIF2α levels in strains with constitutively active CPC-3 (CPC-3^C^). In *cpc-3^C^* cells, P-eIF2α levels are high and arrhythmic (31), despite normal rhythmic PPP-1 levels in the mutant (see Fig. S7 in the supplemental material). This may be due the levels or activity of PPP-1 not being sufficient to dephosphorylate the constantly high levels of P-eIF2α present in this mutant. While we showed that P-eIF2α levels are directly related to PPP-1 levels in a strain with WT CPC-3 activity (Fig. 1B), after 2 h *in vitro* only up to 50% of P-eIF2α was dephosphorylated by PPP-1 indicating that the dephosphorylation step may be kinetically unfavorable (Fig. 1C). These data are consistent with the slow *in vitro* dephosphorylation rate of eIF2α observed in yeast extracts (45). A second possibility for why PPP-1 rhythms are not sufficient to drive P-eIF2α rhythms in the *cpc-3^C^* mutant is that PPP-1 may also regulate CPC-3 activity. This idea is supported by the presence of at least two phosphatases in S. cerevisiae known to target both P-eIF2α and P-GCN2. The 2A-related phosphatase SIT4, which responds to the Target of Rapamycin (TOR) pathway (54, 55) and dephosphorylates eIF2α (56), also controls Ser577 phosphorylation and activity of GCN2. The phosphatase PPZ1 also impacts GCN2-dependent phosphorylation of eIF2α by an unknown mechanism (57, 58). Thus, in addition to direct dephosphorylation of eIF2α, these data support a role for phosphatases controlling the activity of the eIF2α kinases. Experiments are under way to identify potentially rhythmic phosphorylation sites on CPC-3 that may be dephosphorylated by PPP-1. The presence of WT PPP-1 was necessary for dephosphorylation of P-eIF2α *in vitro*. Given the residual *in vitro* phosphatase activity in *eIF2γ^Δ2-62^* cells (Fig. 3B), we cannot absolutely rule out that other PPP-1-dependent phosphatases in the extracts perform the dephosphorylation of P-eIF2α. This residual activity may also explain why the rhythms of P-eIF2α levels are severely diminished, and not completely abolished, in *eIF2γ^Δ2-62^* cells (see Fig. S4). In any case, our data support that PPP-1 is recruited to P-eIF2α by the eIF2 subunit eIF2γ to directly dephosphorylate P-eIF2α.

10.1128/mBio.00871-21.8 FIG S7 PPP-1 protein levels remain rhythmic in *cpc-3^c^* cells, and constitutive activation of CPC-3 does not alter PPP-1 levels. (A) Luciferase activity from a PPP-1::LUC translational fusion expressed in WT (black line) and *cpc-3^c^* (grey line) cells grown in DD and recorded every 90 min over 6 days (Hrs DD). The average normalized bioluminescence signal is plotted (mean ± the SEM, *n *= 12). (B) Western blots of protein from WT and *cpc-3*^c^ cells harvested at DD28 were probed with anti-PPP-1 antibodies. Membranes were stained with amido black as a protein loading control. The graph below shows the average signal of PPP-1 (mean ± the SEM, *n* = 3). Download FIG S7, JPG file, 0.3 MB. Copyright © 2021 Ding et al. 2021 Ding et al. https://creativecommons.org/licenses/by/4.0/ This content is distributed under the terms of the Creative Commons Attribution 4.0 International license.

Kinases typically target specific substrates; however, phosphatases generally have a wide substrate range (45). In addition to dephosphorylation of eIF2α, S. cerevisiae Glc7, the catalytic subunit of PP1, dephosphorylates substrates that function in glycogen metabolism, glucose regulation, and cell division (59). Furthermore, PP1 requires one or more noncatalytic regulatory subunits to target it to different cellular compartments and for substrate specificity. More than 180 PP1 regulatory subunits have been identified in mammalian cells (60), and 17 regulatory subunits were discovered in S. cerevisiae (61). Most, but not all, PP1 regulatory subunits contain a conserved RVxF motif, which is typically flanked by basic residues at the N terminus, and by acidic residues at the C terminus (62). Regulatory subunits that recruit PP1 to eIF2α in mammalian cells, GADD34 and CReP, contain an RVxF motif (43, 44). In the PP1 regulatory subunit eIF2γ in S. cerevisiae, the RVxF motif is present in an N-terminal domain that extends beyond homology to mammalian eIF2γ (45), and deletion of the N terminus of eIF2γ does not affect yeast cell growth, indicating that the eIF2 complex is functional in translation (63). Although N. crassa eIF2γ lacks the conserved RVxF motif (see Fig. S3A), we show that the N terminus of eIF2γ is important for P-eIF2α levels (Fig. 3A), *in vitro* dephosphorylation (Fig. 3B), and rhythmicity (Fig. 3C). Because the levels of eIF2γ are not clock-controlled (Fig. 4B), we suggest that the interaction between the eIF2γ and eIF2α in the eIF2 complex provides a platform for eIF2γ to deliver PPP-1 at night, when it is at peak levels under the control of the clock (Fig. 4A). Furthermore, our data support the possibility that interactions between PPP-1 and eIF2, including eIF2γ and eIF2α subunits, as well as CPC-3, may be localized to the ribosome (see Fig. S2C), although additional experiments are needed to confirm this possibility.

Disruption of P-eIF2α rhythms, either by deletion or mutation of CPC-3 kinase in N. crassa, impacts the rhythmic translation of *alg-11*, but not FRQ (31) or PPP-1 (see Fig. S6) protein rhythms, or overt developmental rhythms (31). These data support that under constant environmental conditions, circadian translational regulation by the rhythmic activity of eIF2α is gene specific, as opposed to a global translational response (31). In *ppp-1^RIP^*cells, the period of FRQ::LUC accumulation rhythms is shorter compared to WT cells (47) (Fig. 2E). However, the short period FRQ::LUC rhythm in *ppp-1^RIP^* is not due to loss of P-eIF2α rhythms in the mutant because disruption of P-eIF2α rhythms in *eIF2γ^Δ2-62^* cells did not significantly alter the period of FRQ::LUC rhythmicity (Fig. 3E).

eIF2α phosphorylation regulates protein production to enable the organism to quickly respond to environmental stresses, including amino acid starvation. The circadian clock provides an additional layer of regulation of eIF2α activity to control the rhythmic translation of specific target genes. While the mechanisms underlying this specificity are not known, these data support the idea that temporal control of eIF2α activity provides organisms, from fungi to mammals, the ability to respond and adapt to internal and environmental stimuli (64). Because mRNA translation requires significant cellular energy, clock control of translation may provide a mechanism to coordinate energy metabolism with translation to partition translation to the times of day when energy levels are high.

## MATERIALS AND METHODS

### N. crassa strains and growth conditions.

N. crassa vegetative growth conditions, transformation and crossing protocols were as described previously (65). Strains generated for use in this study are described in the supplemental materials and methods (see Text S1) and are listed in Table S1 in the supplemental material. The primers used in the generation and validation strains are listed in Table S2.

10.1128/mBio.00871-21.1 TEXT S1 Supplemental materials and methods. Download Text S1, DOCX file, 0.03 MB. Copyright © 2021 Ding et al. 2021 Ding et al. https://creativecommons.org/licenses/by/4.0/ This content is distributed under the terms of the Creative Commons Attribution 4.0 International license.

10.1128/mBio.00871-21.9 TABLE S1 N. crassa strains used in this study. Download Table S1, DOCX file, 0.02 MB. Copyright © 2021 Ding et al. 2021 Ding et al. https://creativecommons.org/licenses/by/4.0/ This content is distributed under the terms of the Creative Commons Attribution 4.0 International license.

10.1128/mBio.00871-21.10 TABLE S2 Primers used in this study. Download Table S2, DOCX file, 0.02 MB. Copyright © 2021 Ding et al. 2021 Ding et al. https://creativecommons.org/licenses/by/4.0/ This content is distributed under the terms of the Creative Commons Attribution 4.0 International license.

### Circadian time courses.

Circadian time course experiments for Western blots were done as previously described (65). For constitutive expression of *bar*::P*tcu-1*::*ppp-1*, cells were grown in Vogel’s medium containing 50 μM the copper chelator bathocuproinedisulfonic acid (BCS, B1125; Sigma-Aldrich, St. Louis, MO) or 250 μM copper sulfate (CuSO_4_; C7631; Sigma-Aldrich) to control the expression of the *tcu-1* promoter (48).

### Protein extraction and Western blotting.

Protein extraction, protein concentration, and Western blot analyses were performed as previously described (28). Briefly, tissue was ground in liquid nitrogen with a mortar and pestle, and suspended in extraction buffer containing 100 mM Tris pH 7.0, 1% sodium dodecyl sulfate (SDS), 10 mM NaF, 1 mM phenylmethylsulfonyl fluoride, 1 mM sodium ortho-vanadate, 1 mM β-glycerophosphate, 1× aprotinin, 1× leupeptin hemisulfate salt, and 1× pepstatin A. Protein concentration was determined by NanoDrop (Thermo Fisher Scientific, Wilmington, DE). Protein samples (100 μg) were separated on 10% SDS-PAGE gels and blotted to Immobilon-P nitrocellulose membranes (catalog no. IPVH00010; Millipore Sigma, Burlington, MA) according to standard methods.

The levels of P-eIF2α were detected using rabbit monoclonal anti-EIF2S1 (phospho S51) antibody (catalog no. ab32157; Abcam, Cambridge, UK) diluted 1:5000 in 5% bovine serum albumin, 1× Tris-buffered saline (TBS), 0.1% Tween, and anti-rabbit IgG horseradish peroxidase (HRP)-conjugated secondary antibody (catalog no. 1706515; Bio-Rad, Hercules, CA) diluted 1:10,000. Total eIF2α levels were detected using rabbit polyclonal anti-EIF2S1 antibody (catalog no. 47508; Abcam) diluted 1:5,000, and anti-rabbit IgG HRP secondary antibody diluted 1:10,000. eIF2γ::V5 was detected using mouse monoclonal anti-V5 antibody (catalog no. R960-25; Invitrogen, Carlsbad, CA) diluted 1:5,000 in 5% milk, 1× TBS, 0.1% Tween, and anti-mouse IgG HRP-secondary antibody (catalog no. 1706516; Bio-Rad) diluted 1:10,000. PPP-1 was detected using a custom rabbit polyclonal anti-PPP-1 antibody (peptide EVRGSRPGKQVQLLC as antigen; GenScript, Piscataway, NJ) diluted 1:1,000 in 7.5% milk, 1× TBS, 0.1% Tween, and anti-rabbit IgG HRP-secondary antibody diluted 1:10,000. Signals were detected using chemiluminescence SuperSignal West Pico substrate (catalog no. 34077; Thermo Fisher Scientific). Densitometry was performed using NIH ImageJ software (66) and normalized to protein loading using amido black-stained protein.

### Expression and purification of PPP-1::His6 protein in *E. coli.*

To validate the specificity of PPP-1 antibody, the *ppp-1* ORF was amplified with the primers PPP-1::His6 F and PPP-1::His6 R containing restriction sites for NdeI and NotI using N. crassa cDNA as the template. The pET30b vector (Invitrogen) and PCR fragment were digested with NdeI and NotI restriction enzymes and then ligated with T7 ligase (NEB). The ligated plasmids were transformed to E. coli DH5α cells and screened by kanamycin resistance and restriction digestion to get an IPTG (isopropyl-β-d-thiogalactopyranoside)-inducible PPP-1::His6 fusion plasmid. The plasmid was transformed into E. coli BL21 cells and grown in 400 ml of Luria-Bertani medium at 37°C with shaking at 250 rpm to an optical density of 0.6. PPP-1::His6 expression was induced by adding 1 mM IPTG 1 h before protein extraction. PPP-1::His6 protein was purified with Ni-NTA column following published methods (67). PPP-1::His6 protein was visualized by Coomassie blue stain and Western blotting with PPP-1 antibody.

### *In vivo* luciferase assays.

Luciferase assays to examine bioluminescence rhythms arising from strains containing luciferase fusions were performed as previously described (28). Briefly, 5 μl of 1 × 10^5^ conidia/ml were inoculated into 96-well microtiter plates containing 150 μl of 1× Vogel’s salts, 0.01% glucose, 0.03% arginine, 0.1 M quinic acid, 1.5% agar, and 25 μM firefly luciferin (LUNCA-300; Gold Biotechnology, St. Louis, MO) (pH 6). After inoculation, the microtiter plate was incubated at 30°C in constant light (LL) for 24 h and transferred to DD 25°C to obtain bioluminescence recordings using an EnVision Xcite Multilabel Reader (Perkin-Elmer Life Science, Boston, MA), with recordings taken every 90 min over at least 5 continuous days. Raw luciferase activity data were analyzed for period using BioDARE (68). Raw reads were normalized to the mean to graph the data.

### Statistical analysis.

Circadian time course data were examined using F tests of the fit of the data to a sine wave or a line, as previously described (65, 69). A Student's *t*-test was used to determine significance in changes in the levels of P-eIF2α and PPP-1. Error bars in all graphs represent the standard errors of the mean (SEM) from at least three independent experiments.

### *In vitro* dephosphorylation assay.

The eIF2 complex was isolated by anti-V5 coimmunoprecipitation from an eIF2γ::V5 and eIF2γ^Δ2-62^::V5 protein extracts. The eIF2 complex was immobilized onto magnetic Dynabeads (catalog no. 10008D; Invitrogen) and washed with 2× phosphatase buffer (100 mM HEPES, 200 mM NaCl, 2 mM dithiothreitol, 2 mM MnCl_2_, 0.01% Brij-35) (45). Then, 500 μg of protein extracted from Δ*cpc-3* or *ppp-1^RIP^*; Δ*cpc-3* or *eIF2γ^Δ2-62^*; *Δcpc-3* strains, harvested at DD28, was mixed with 200 μl of the immobilized eIF2-Dynabeads in 2× phosphatase buffer. Reaction mixtures were incubated at 30°C with gentle rotation, and at each time point 48 μl of the reaction mix was transferred to a fresh tube and boiled for 5 min with 16 μl of 4× SDS loading buffer (250 mM [pH 6.8] Tris-Cl, 8% SDS, 0.2% bromophenol blue, 40% glycerol, 20% β-mercaptoethanol) to stop the reaction. P-eIF2α and total eIF2α levels were detected by Western blotting.

### Sucrose gradient fractionation.

Linear sucrose gradients (10 to 50% in 10 mM HEPES-KOH, 70 mM ammonium acetate, 5 mM magnesium acetate) were prepared in ultracentrifuge tubes by using a BIOCOMP gradient station (Fredericton, NB, Canada) and stored at 4°C before use. Extracts were prepared by adding polysome extraction buffer (100 mM KCl, 20 mM HEPES-KOH, 10 mM magnesium acetate, 15 mM β-mercaptoethanol, 100 μg/ml cycloheximide) to ground tissues and centrifuging the solution to remove cellular debris and lipids. Next, 400 μl of the extract containing 100 *A*_260_ units/ml (1 *A*_260_ unit corresponds to an absorbance of 1.0 at 260 nm) was added onto the sucrose gradient and centrifuged at 41,000 rpm for 2h at 4°C. The samples were then divided into 14 fractions of approximately 1 ml each using the BIOCOMP. The absorbances at 260 nm were used as a proxy for RNA content and graphed against the fraction of the gradient. Disome, trisome, tetrasome, and pentosome fractions were pooled as the polysome fraction. Fractions representing the 40S (#4), 60S (#5), 80S (#6) ribosome and the pooled polysome fraction were boiled in SDS loading buffer (250 mM [pH 6.8] Tris-Cl, 8% SDS, 0.2% bromophenol blue, 40% glycerol, 20% β-mercaptoethanol), and 15 μl was separated on a 10% SDS-PAGE gel for Western blotting.
