# Supplementary figures and images for: Circadian Clock Control of Translation Initiation Factor eIF2α Activity Requires eIF2γ-Dependent Recruitment of Rhythmic PPP-1 Phosphatase in Neurospora crassa
Source: mBio. 2021 May 18;12(3):e00871-21. doi: 10.1128/mBio.00871-21 (PMC8262944; doi:10.1128/mBio.00871-21)

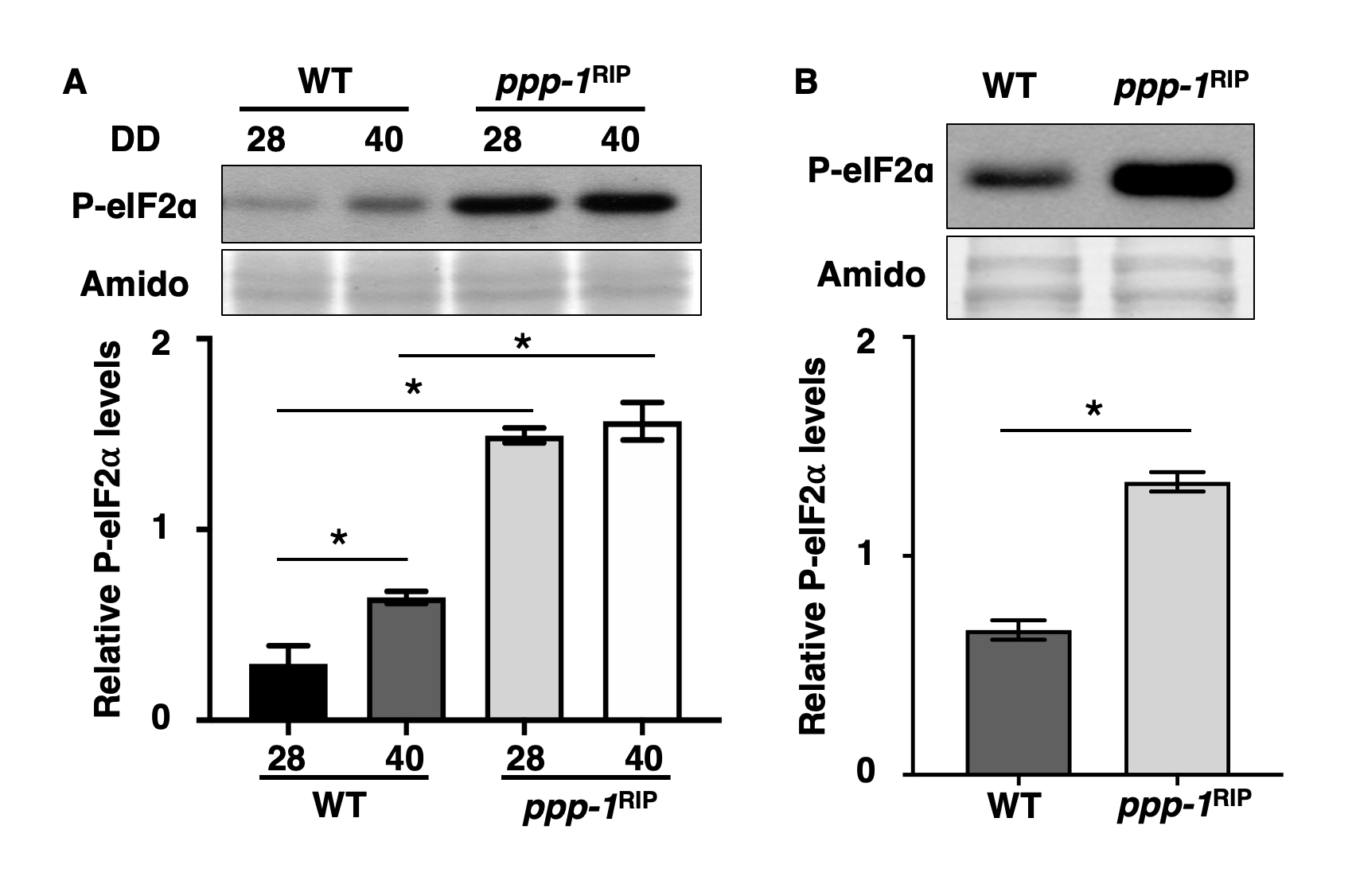

Supplement: FIG S1 [file mbio.00871-21-sf001.jpg]

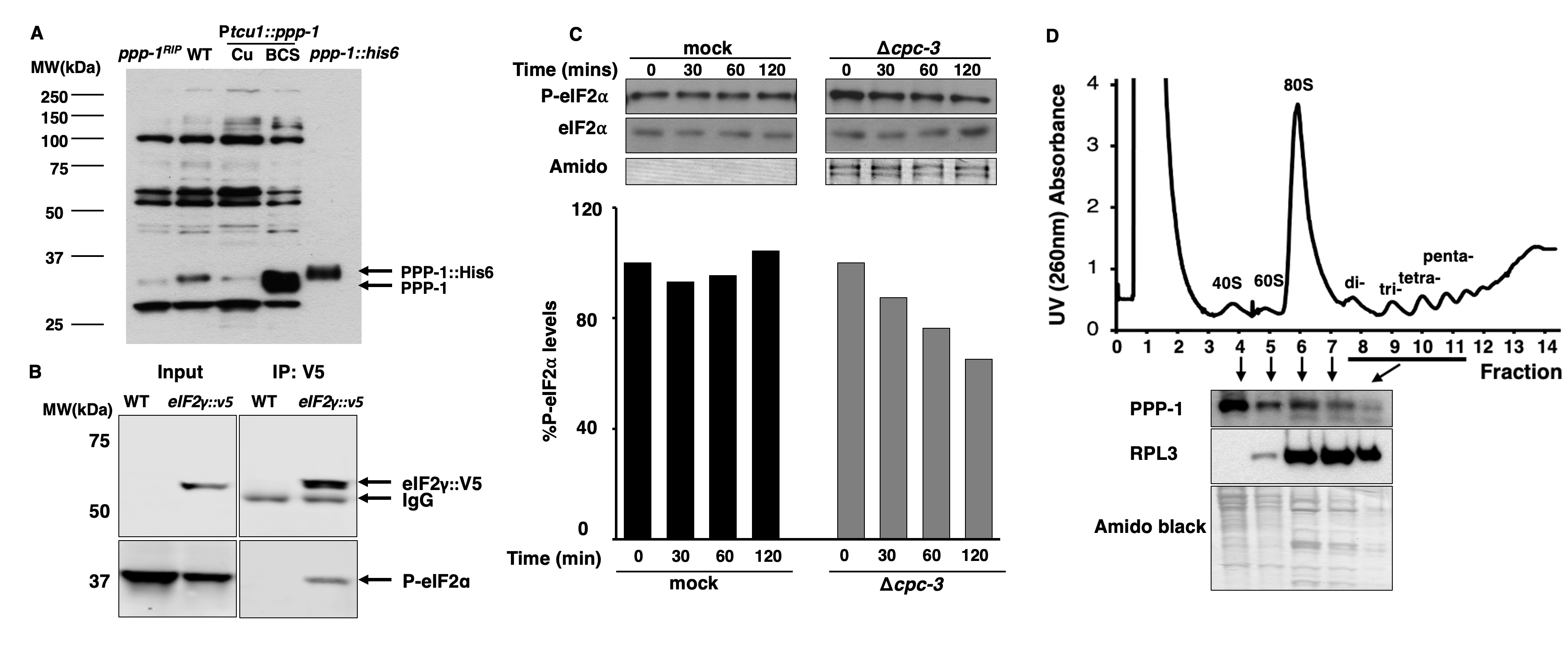

Supplement: FIG S2 [file mbio.00871-21-sf002.jpg]

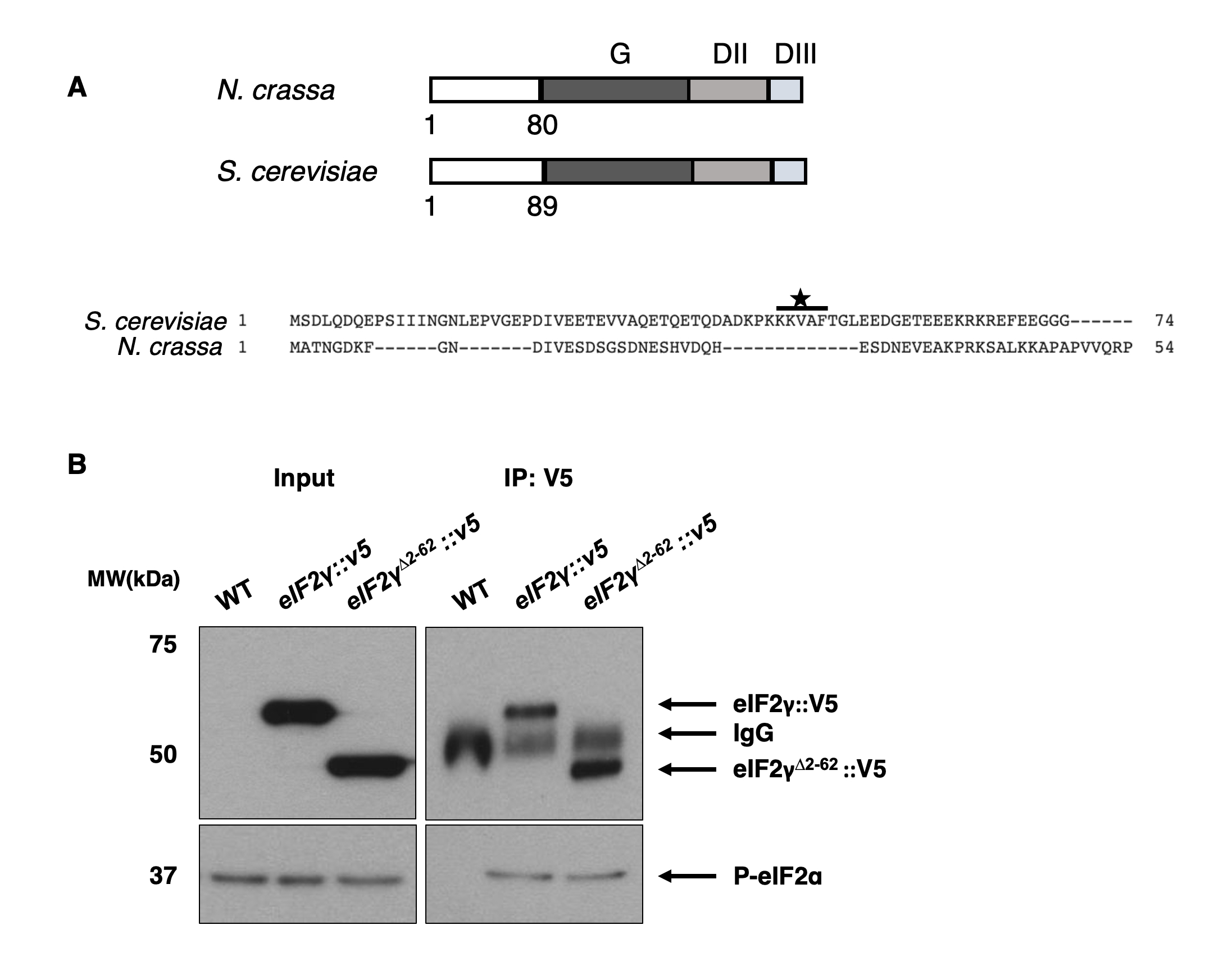

Supplement: FIG S3 [file mbio.00871-21-sf003.jpg]

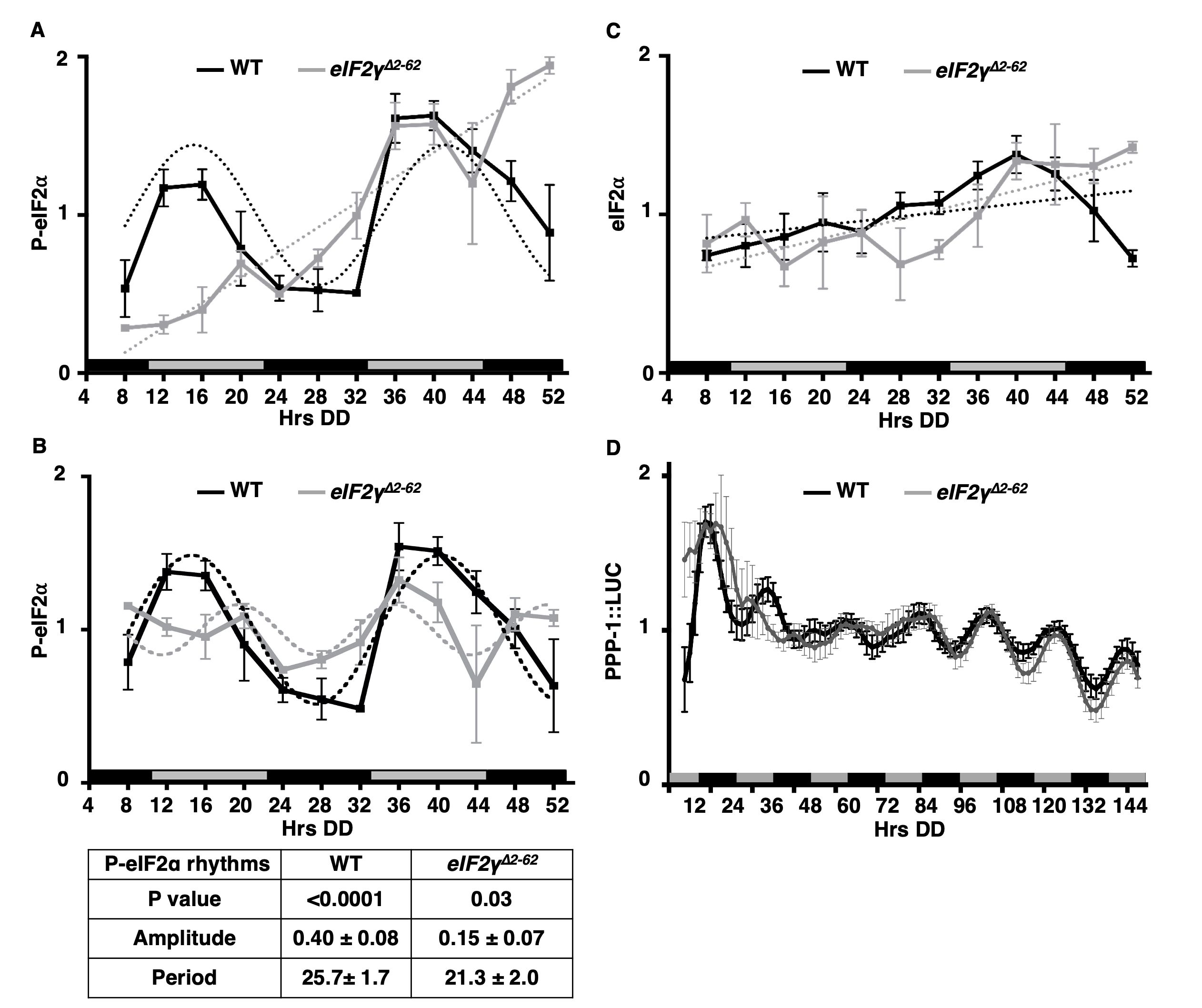

Supplement: FIG S4 [file mbio.00871-21-sf004.jpg]

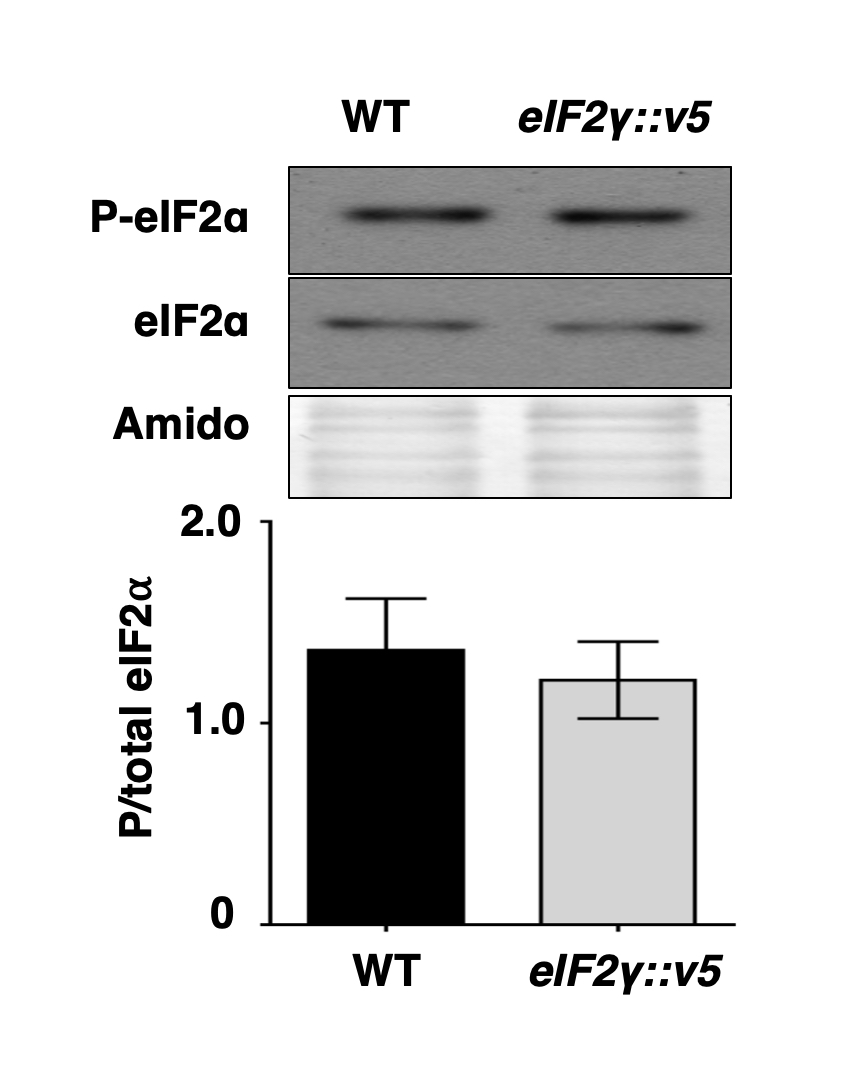

Supplement: FIG S5 [file mbio.00871-21-sf005.jpg]

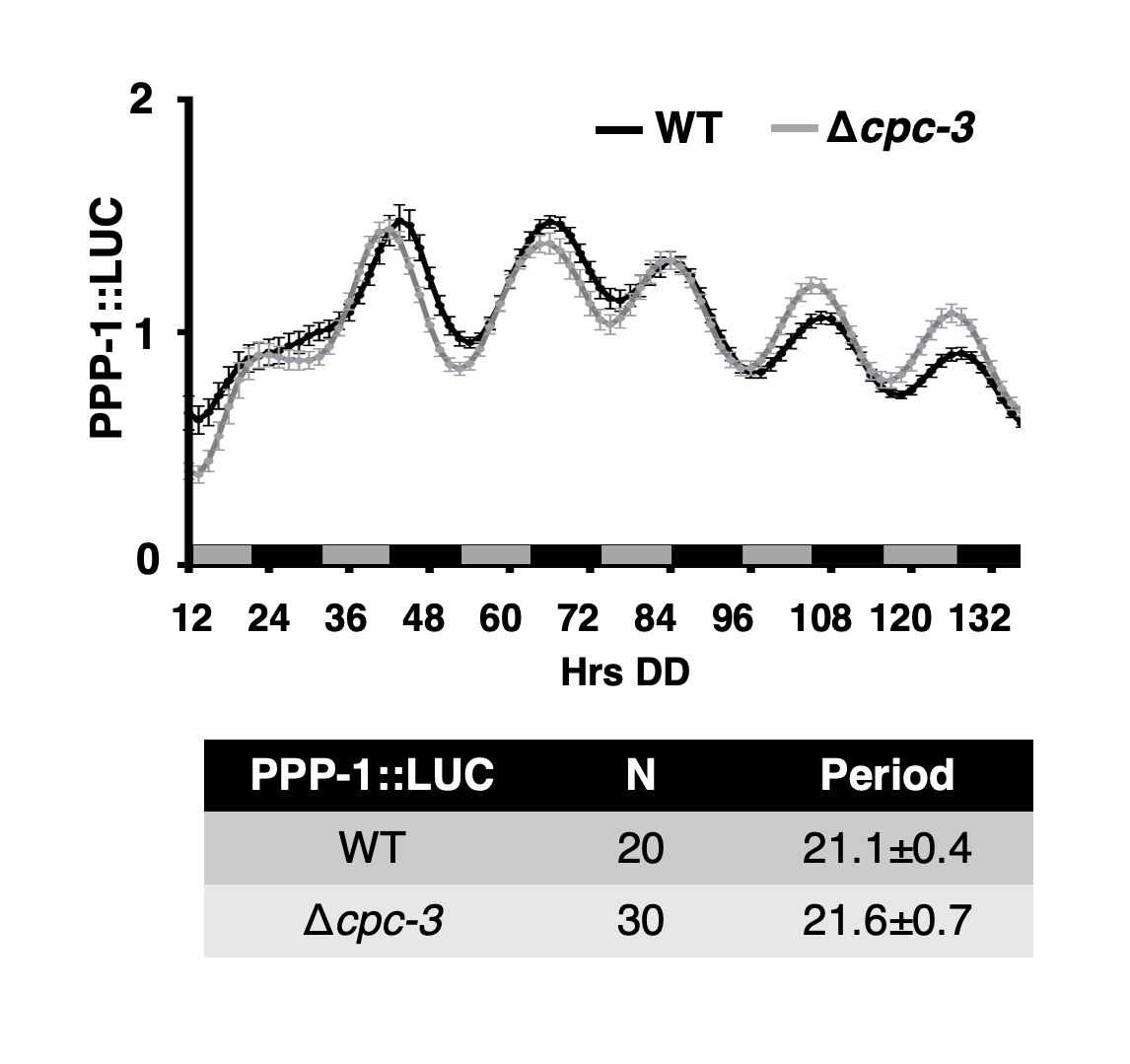

Supplement: FIG S6 [file mbio.00871-21-sf006.jpg]

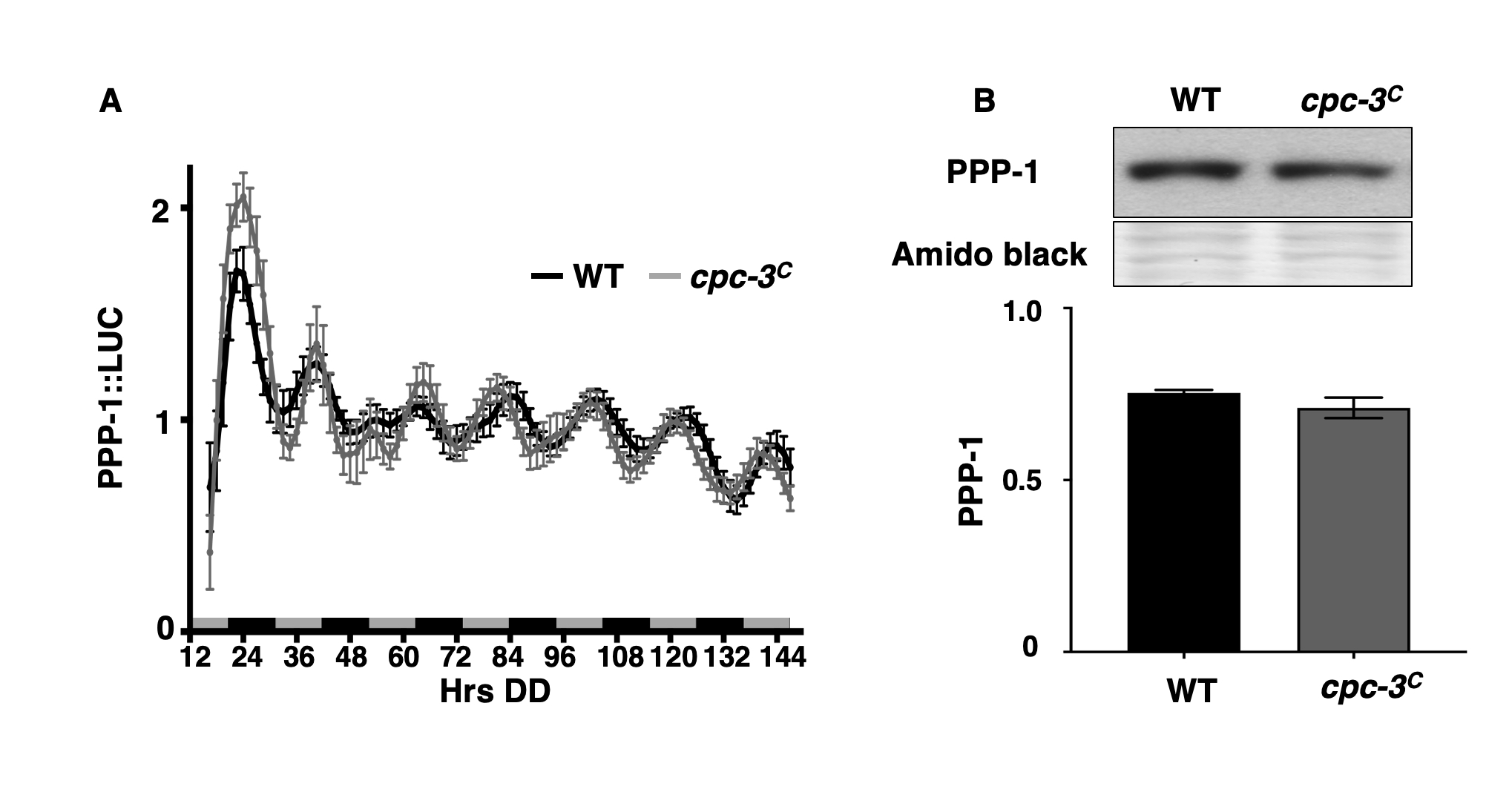

Supplement: FIG S7 [file mbio.00871-21-sf007.jpg]
