# Supplementary material for: Circadian Clock Control of Translation Initiation Factor eIF2α Activity Requires eIF2γ-Dependent Recruitment of Rhythmic PPP-1 Phosphatase in Neurospora crassa
Source: mBio. 2021 May 18;12(3):e00871-21. doi: 10.1128/mBio.00871-21 (PMC8262944; doi:10.1128/mBio.00871-21)
Supplement: TEXT S1 [file mbio.00871-21-s0001.docx]

**SI Materials and Methods**

***N. crassa* strains and growth conditions.** The strains used in this study are listed in **Table S1**. Strains with the hph cassette were maintained on Vogel’s minimal media containing 0.2 g/L of hygromycin B (#80055-286, VWR, Radner, PA). Strains with the bar cassette were maintained on Vogel’s minimal media without NH_4_NO_3_ and with 0.5% proline and 0.2 g/L BASTA (Liberty 280 SL Herbicide, Bayer, NC).

*N. crassa* wild type (WT) FGSC #4200 (mat a, 74-OR23-IV) or FGSC #2489 (mat A, 74-OR23-IV), were obtained from the Fungal Genetics Stock Center (FGSC, Kansas State University). DBP 1228 (∆*frq::bar*) (1) and DBP 3291 (*cpc-3^c^*) (2) were previously generated. The primers used in the generation and validation strains are listed in **Table S2**. DBP3070 (*ppp-1^RIP^*) was generated by crossing *ppp-1^RIP^*, *ras-1^bd^,* which was kindly provided by Dr. Yi Liu (3), with WT (FGSC #2489). Progeny were screened by their slow growth phenotype, and validated by PCR using *ppp-1* seq F1 and R1 primers, followed by DNA sequencing using primers *ppp-1* seq F. To complement *ppp-1^RIP^*, the *ppp-1* gene was inserted into the *csr-1* locus by homologous recombination using a product from 3-way PCR containing 1kb of the *csr-1* 5’flank (primers *ppp-1* F1 and R1), the *ppp-1* gene with the 5’ and 3’ UTRs (primers *ppp-1* F2 and R2), and 1kb of the *csr-1* 3’flank (primers *ppp-1* F3 and R3) to generate *ppp-1^RIP^*; *csr-1::ppp-1* (DBP3457). The 3-way PCR product was transformed into *ppp-1^RIP^* (DBP3070) and primary transformants were selected by cyclosporine A (30024-25MG, Sigma-Aldrich St. Louis, MO) resistance (5 µg/ml), and validated by PCR using *ppp-1* F and R. For overexpression of PPP-1, the *bar::Ptcu-1::ppp-1* strain was generated by transforming a 3-way PCR product containing 1 kb of the 5’ *ppp-1* ORF (primers tcu1 F1 and R1), *bar::*P*tcu-1* from plasmid pDBP450 (primers tcu1 F2 and R2) (4), and 1 kb of the 3’ end of *ppp-1* (primers tcu1 F3 and R3) into the WT (FGSC #4200) strain. Homologous recombination was validated by PCR (primers tcu1 F4 and R4), and a *bar::*P*tcu-1::ppp-1* homokaryon (DBP3279) was obtained by microconidia filtration (5). To generate strains deleted for the eIF2γ N-terminus (2-62 aa) at the endogenous locus, the *eIF2γ*^∆2-62^ aa fragment was generated by 2-way PCR using primers *eIF2γ^∆2-62^* F1, R1, F2 and R2. The PCR product was co-transformed with hyg^R^ pBP15 plasmid (6) into ∆*mus-52::bar* (FGSC#9719). Homologous recombinants were screened by PCR (primers *eIF2γ^∆2-62^* F3 and R3), and selected based on the size of the PCR product. A transformant containing *eIF2γ^∆2-62^* was crossed to WT (FGSC#2489) to obtain an *eIF2γ^∆2-62^* homokaryon (DBP3297). To assay FRQ::LUC luciferase activity, strains *ppp-1^RIP^* (DBP3070) and *eIF2γ^∆2-62^* (DBP3297) were crossed to DBP1563 containing a FRQ::LUC translational fusion linked to *bar* (7), respectively. Progeny were screened for luciferase activity arising from *frq::luc*, sequencing of *ppp-1^RIP^*, or PCR for *eIF2γ^∆2-62^*, to obtain *frq::luc*, *ppp-1^RIP^* (DBP3356) and *frq::luc*, *eIF2γ^∆2-62^* (DBP3424). The PPP-1::LUC translational fusion at the endogenous locus was generated by 3-way PCR of the PPP-1 coding region (primers *ppp-1::luc* F1 and R1), *N. crassa* codon-optimized luciferase gene (8) (primers *ppp-1::luc* F2 and R2) and PPP-1 3’ flank (primers *ppp-1::luc* F3 and R3). The 3-way PCR fragments were co-transformed with hyg^R^ pBP15 into ∆*mus-52::bar* (FGSC#9719) and screened by luciferase activity and PCR (primers *ppp-1::luc* F4 and R4) to confirm homologous insertion into the *ppp-1* gene. A transformant containing *ppp-1::luc* was crossed with WT (FGSC #2489) to get the homokaryon. *ppp-1::luc* was crossed to *∆frq::bar* (DBP1228), ∆*cpc-3* (FGSC #10697), *cpc-3^c^* (DBP3291) and *eIF2γ^∆2-62^* (DBP3297) strains to generate a *ppp-1::luc* (DBP2889), *ppp-1::luc, ∆frq* (DBP3001), *ppp-1::luc, ∆cpc-3* (DBP3368), *ppp-1::luc, cpc-3^c^* (DBP3837) and *ppp-1::luc, eIF2γ^∆2-62^* (DBP3836) strains respectively. *eIF2γ::v5* and *eIF2γ^∆2-62^::v5* endogenously tagged strains were generated by 3-way PCR with the *eIF2γ* ORF region (primers *eIF2γ::v5* F1 and R1), *v5* gene from plasmid pDBP525 (primers *eIF2γ::v5* F2 and R2) and the 3’ end of *eIF2γ* (primers *eIF2γ::v5* F3 and R3). The 3-way PCR product was co-transformed with hyg^R^ pBP15 plasmid into ∆*mus-52::bar* (FGSC#9719) and *eIF2γ^∆2-62^* (DBP3297), respectively. Homologous recombinants were screened by western blot using anti-V5 antibody, and by PCR (*eIF2γ::v5* F4 and R4) to verify homologous insertion of *v5* at *eIF2γ* and *eIF2γ^∆2-62^*. A transformant with *eIF2γ::v5* and *eIF2γ^∆2-62^::v5* was crossed with WT (FGSC #2489) and screened by PCR (*eIF2γ::v5* F5 and R5) to obtain an *eIF2γ::v5* (DBP3428) and *eIF2γ^∆2-62^::v5* (DBP3706) homokaryon. The *ppp-1^RIP^; ∆cpc-3* (DBP3533) strain was generated by crossing *ppp-1^RIP^* (DBP3070) with *∆cpc-3* (DBP1883) cells, and screened for *∆cpc-3* by PCR as described previously (2), and *ppp-1^RIP^* as described above. The *eIF2γ^∆2-62^; ∆cpc-3* (DBP3657) strain was generated by crossing *eIF2γ^∆2-62^* (DBP3298) with *∆cpc-3* (DBP1883). and screened for *∆cpc-3* by PCR (2) and *eIF2γ^∆2-62^*.

**References**

1. Bennett LD, Beremand P, Thomas TL, Bell-Pedersen D. 2013. Circadian activation of the mitogen-activated protein kinase MAK-1 facilitates rhythms in clock-controlled genes in *Neurospora crassa*. Eukaryot Cell 12:59-69.

2. Karki S, Castillo K, Ding Z, Kerr O, Lamb TM, Wu C, Sachs MS, Bell-Pedersen D. 2020. Circadian clock control of eIF2α phosphorylation is necessary for rhythmic translation initiation. Proc Natl Acad Sci U S A 117:10935-10945.

3. Yang Y, He Q, Cheng P, Wrage P, Yarden O, Liu Y. 2004. Distinct roles for PP1 and PP2A in the Neurospora circadian clock. Genes Dev 18:255-60.

4. Larrondo LF, Loros JJ, Dunlap JC. 2012. High-resolution spatiotemporal analysis of gene expression in real time: *in vivo* analysis of circadian rhythms in *Neurospora crassa* using a FREQUENCY-luciferase translational reporter. Fungal Genet Biol 49:681-3.

5. Lamb TM, Vickery J, Bell-Pedersen D. 2013. Regulation of gene expression in *Neurospora crassa* with a copper responsive promoter. G3 (Bethesda) 3:2273-80.

6. Ebbole DJ, and Sachs, M.S. 1990. A rapid and simple method for isolation of *Neurospora crassa* homokaryons using microconidia. Fungal Genetics Newsletter 37:17-18.

7. Beasley A, Lamb TM, Versaw W, Bell-Pedersen D. 2006. A *ras-1^bd^* Mauriceville strain for mapping mutations in Oak Ridge *ras-1^bd^* strains. Fungal Genetics Reports 53(9):30-33.

8. Gooch VD, Mehra A, Larrondo LF, Fox J, Touroutoutoudis M, Loros JJ, Dunlap JC. 2008. Fully codon-optimized luciferase uncovers novel temperature characteristics of the Neurospora clock. Eukaryot Cell 7:28-37.
