# Supplementary material for: Circadian Clock Control of Translation Initiation Factor eIF2α Activity Requires eIF2γ-Dependent Recruitment of Rhythmic PPP-1 Phosphatase in Neurospora crassa
Source: mBio. 2021 May 18;12(3):e00871-21. doi: 10.1128/mBio.00871-21 (PMC8262944; doi:10.1128/mBio.00871-21)
Supplement: TABLE S1 [file mbio.00871-21-st001.docx]

**Table S1. *N. crassa* strains used in this study**

| Name | Description | Source |
| --- | --- | --- |
| FGSC4200 | *Neurospora crassa* wild type 74-OR23-IV mat a | FGSC |
| FGSC2489 | *Neurospora crassa* wild type 74-OR23-IV mat A | FGSC |
| FGSC9719 | ∆*mus-52::bar*, mat a | FGSC |
| DBP1228 | ∆*frq::bar*, mat a | (1) |
| DBP3291 | *cpc-3*^c^ | (2) |
| DBP3007 | *ppp-1^RIP^*, *ras-1^bd^* | (3) |
| DBP3070 | *ppp-1^RIP^* | This study |
| DBP3457 | *ppp-1^RIP^*; *csr-1::ppp-1* | This study |
| DBP3279 | *bar::*P*_tcu-1_::ppp-1*, mat a | This study |
| DBP3297 | *eIF2γ*^∆2-62^ | This study |
| DBP1563 | *frq::luc::bar* translation fusion | (4) |
| DBP3356 | *frq::luc*, *ppp-1^RIP^* | This study |
| DBP3424 | *frq::luc*, *eIF2γ^∆2-62^* | This study |
| DBP2889 | *ppp-1::luc* | This study |
| DBP3001 | *ppp-1::luc, ∆frq* | This study |
| DBP3428 | *eIF2γ::v5* | This study |
| DBP3706 | *eIF2γ^∆2-62^::v5* | This study |
| DBP3533 | *ppp-1^RIP^; ∆cpc-3* | This study |
| FGSC10697 | *∆cpc-3::hph,* mat a | FGSC |
| DBP3657 | *eIF2γ^∆2-62^; ∆cpc-3* | This study |
| DBP3368 | *ppp-1::luc, ∆cpc-3* | This study |
| DBP3837 | *ppp-1::luc, cpc-3^c^* | This study |
| DBP3836 | *ppp-1::luc, eIF2γ^∆2-62^* | This study |

**Table S1 References**

1. Bennett LD, Beremand P, Thomas TL, Bell-Pedersen D. 2013. Circadian activation of the mitogen-activated protein kinase MAK-1 facilitates rhythms in clock-controlled genes in *Neurospora crassa*. Eukaryot Cell 12:59-69.

2. Karki S, Castillo K, Ding Z, Kerr O, Lamb TM, Wu C, Sachs MS, Bell-Pedersen D. 2020. Circadian clock control of eIF2alpha phosphorylation is necessary for rhythmic translation initiation. Proc Natl Acad Sci U S A 117:10935-10945.

3. Yang Y, He Q, Cheng P, Wrage P, Yarden O, Liu Y. 2004. Distinct roles for PP1 and PP2A in the *Neurospora* circadian clock. Genes Dev 18:255-60.

4. Larrondo LF, Loros JJ, Dunlap JC. 2012. High-resolution spatiotemporal analysis of gene expression in real time: *in vivo* analysis of circadian rhythms in *Neurospora crassa* using a FREQUENCY-luciferase translational reporter. Fungal Genet Biol 49:681-3.
