# Supplementary material for: Circadian Clock Control of Translation Initiation Factor eIF2α Activity Requires eIF2γ-Dependent Recruitment of Rhythmic PPP-1 Phosphatase in Neurospora crassa
Source: mBio. 2021 May 18;12(3):e00871-21. doi: 10.1128/mBio.00871-21 (PMC8262944; doi:10.1128/mBio.00871-21)
Supplement: TABLE S2 [file mbio.00871-21-st002.docx]

**Table S2. Primers used in this study**

| **Primer name** | **Used for** | **Primer sequence (5'->3' orientation)** |
| --- | --- | --- |
| *ppp-1* seq F1 | Sequence *ppp-1^RIP^* | GCTTTGCAGAAGTGCATCTAC |
| *ppp-1* seq R1 | Sequence *ppp-1^RIP^* | GACGTTTATCGGCGGCCGAAC |
| *ppp-1* seq F | Sequence *ppp-1^RIP^* | ATGGCGGACCATACCGAAGTC |
| *ppp-1* F1 | *ppp-1^RIP^* complementation | CGCTGGAGAAGCTCATTCCAC |
| *ppp-1* R1 | *ppp-1^RIP^* complementation | GAAACGCACAGGCACCTCAATGTCCATCTGATC |
| *ppp-1* F2 | *ppp-1^RIP^* complementation | CATTGAGGTGCCTGTGCGTTTCATTAGCTGC |
| *ppp-1* R2 | *ppp-1^RIP^* complementation | CTGTCTAGGCTTCGAGAACCACCCTCTATC |
| *ppp-1* F3 | *ppp-1^RIP^* complementation | GTTCTCGAAGCCTAGACAGTTTGCAGGTCATGTG |
| *ppp-1* R3 | *ppp-1^RIP^* complementation | CAACCGAGCACACCAACTAAC |
| *ppp-1* F4 | *ppp-1^RIP^* complementation | CTGCTACCGCCATACGAAGTG |
| *ppp-1* R4 | *ppp-1^RIP^* complementation | GCAAAGAAGTGCGTTACTACC |
| tcu1 F1 | P*tcu1::ppp-1* | GTAAGGTGTCACACTCACAGTC |
| tcu1 R1 | P*tcu1::ppp-1* | TTAGGTCGACGCAGCTAATGAAACGCACAGG |
| tcu1 F2 | P*tcu1::ppp-1* | CATTAGCTGCGTCGACCTAAATCTCGGTGAC |
| tcu1 R2 | P*tcu1::ppp-1* | GGTCCGCCATGGTTGGGGATGTGTGTGCGA |
| tcu1 F3 | P*tcu1::ppp-1* | ATCCCCAACCATGGCGGACCATACCGAAGTC |
| tcu1 R3 | P*tcu1::ppp-1* | GTCGACGCTCATCATAGCACC |
| tcu1 F4 | P*tcu1::ppp-1* | GCTGTTGTAATCATGCATACC |
| tcu1 R4 | P*tcu1::ppp-1* | GAATTGCTTCATCTGAGACTG |
| *eIF2γ^∆2-62^* F1 | *eIF2γ^∆2-62^* | GTTGCAGAGTAACGATTGTTC |
| *eIF2γ^∆2-62^* R1 | *eIF2γ^∆2-62^* | GACGTCGAGGTCCATGATTGTGGATGTGGGTGG |
| *eIF2γ^∆2-62^* F2 | *eIF2γ^∆2-62^* | CCACAATCATGGACCTCGACGTCAAGACCCTC |
|  |  | (continued) |
| *eIF2γ^∆2-62^* R2 | *eIF2γ^∆2-62^* | GAGACAATCCTGCTGAAGATG |
| *eIF2γ^∆2-62^* F3 | *eIF2γ^∆2-62^* | AGTACACAGCAACTACCAGGC |
| *eIF2γ^∆2-62^* R3 | *eIF2γ^∆2-62^* | GCTCATGAGAATATCGTGACC |
| *ppp-1::luc* F1 | PPP-1::LUC | GGTCAATACTATGATCTCCTCC |
| *ppp-1::luc* R1 | PPP-1::LUC | GATGTTCTTGGCGTCCTCCATTCGGCGGCCGAACCTAAAGG |
| *ppp-1::luc* F2 | PPP-1::LUC | CCTTTAGGTTCGGCCGCCGAATGGAGGACGCCAAGAACATC |
| *ppp-1::luc* R2 | PPP-1::LUC | GCGGGCGAAAGTATCAGACGTATCAGACGGCGATCTTGCC |
| *ppp-1::luc* F3 | PPP-1::LUC | GGCAAGATCGCCGTCTGATACGTCTGATACTTTCGCCCGC |
| *ppp-1::luc* R3 | PPP-1::LUC | CTCGAATACAGCATGGTGTTCG |
| *ppp-1::luc* F4 | PPP-1::LUC | GGTAAGCAGGTGCAGCTGCTG |
| *ppp-1::luc* R4 | PPP-1::LUC | CACTCTTGCCTCTCAGTCACC |
| *eIF2γ::V5* F1 | eIF2γ::V5 | CAGCACTACGAGTCCATCCTC |
| *eIF2γ::V5* R1 | eIF2γ::V5 | TCCGCCGCCTCCAGAGGTAGAAGGCTCGAGGGT |
| *eIF2γ::V5* F2 | eIF2γ::V5 | GCCTTCTACCTCT GGAGGCGGCGGAGGCGGTAA |
| *eIF2γ::V5* R2 | eIF2γ::V5 | CTGGTCGCTCGC CTACGTAGAATCGAGACCGAG |
| *eIF2γ::V5* F3 | eIF2γ::V5 | GATTCTACGTAGGCGAGCGACCAGAAAATATCC |
| *eIF2γ::V5* R3 | eIF2γ::V5 | CTATTCGATGTACAGGAGGTG |
| *eIF2γ::V5* F4 | eIF2γ::V5 | CTCGGTCTCGATTCTACGTAG |
| *eIF2γ::V5* R4 | eIF2γ::V5 | GTGATTGAGAATCTGGTGAAGC |
| *eIF2γ::V5* F5 | eIF2γ::V5 | ACCCTCGAGCCTTCTACCTCT |
| *eIF2γ::V5* R5 | eIF2γ::V5 | GATATTCATCATTAGAGCAAC |
| PPP-1::His6 F | PPP-1::His6 | ACGAGTCATATGGCGGACCATACCGAAGTC |
| PPP-1::His6 R | PPP-1::His6 | ATAATCGCGGCCGCTCGGCGGCCGAACTTCTG |
